# Using *C*. *elegans* Forward and Reverse Genetics to Identify New Compounds with Anthelmintic Activity

**DOI:** 10.1371/journal.pntd.0005058

**Published:** 2016-10-18

**Authors:** Mark D. Mathew, Neal D. Mathew, Angela Miller, Mike Simpson, Vinci Au, Stephanie Garland, Marie Gestin, Mark L. Edgley, Stephane Flibotte, Aruna Balgi, Jennifer Chiang, Guri Giaever, Pamela Dean, Audrey Tung, Michel Roberge, Calvin Roskelley, Tom Forge, Corey Nislow, Donald Moerman

**Affiliations:** 1 Department of Zoology and Michael Smith Laboratories, University of British Columbia, Vancouver, British Columbia, Canada; 2 Department of Pharmaceutical Sciences, University of British Columbia, Vancouver, British Columbia, Canada; 3 Universite de Poitiers, Poitiers, France; 4 Department of Biochemistry and Molecular Biology, University of British Columbia, Vancouver, British Columbia, Canada; 5 Department of Cellular and Physiological Sciences, University of British Columbia, Vancouver, British Columbia, Canada; 6 Summerland Research and Development Centre, Agriculture and Agri-Food Canada, Summerland, British Columbia, Canada; McGill University, CANADA

## Abstract

**Background:**

The lack of new anthelmintic agents is of growing concern because it affects human health and our food supply, as both livestock and plants are affected. Two principal factors contribute to this problem. First, nematode resistance to anthelmintic drugs is increasing worldwide and second, many effective nematicides pose environmental hazards. In this paper we address this problem by deploying a high throughput screening platform for anthelmintic drug discovery using the nematode *Caenorhabditis elegans* as a surrogate for infectious nematodes. This method offers the possibility of identifying new anthelmintics in a cost-effective and timely manner.

**Methods/Principal findings:**

Using our high throughput screening platform we have identified 14 new potential anthelmintics by screening more than 26,000 compounds from the Chembridge and Maybridge chemical libraries. Using phylogenetic profiling we identified a subset of the 14 compounds as potential anthelmintics based on the relative sensitivity of *C*. *elegans* when compared to yeast and mammalian cells in culture. We showed that a subset of these compounds might employ mechanisms distinct from currently used anthelmintics by testing diverse drug resistant strains of *C*. *elegans*. One of these newly identified compounds targets mitochondrial complex II, and we used structural analysis of the target to suggest how differential binding of this compound may account for its different effects in nematodes versus mammalian cells.

**Conclusions/Significance:**

The challenge of anthelmintic drug discovery is exacerbated by several factors; including, 1) the biochemical similarity between host and parasite genomes, 2) the geographic location of parasitic nematodes and 3) the rapid development of resistance. Accordingly, an approach that can screen large compound collections rapidly is required. *C*. *elegans* as a surrogate parasite offers the ability to screen compounds rapidly and, equally importantly, with specificity, thus reducing the potential toxicity of these compounds to the host and the environment. We believe this approach will help to replenish the pipeline of potential nematicides.

## Author Summary

With over two billion people infected and many billions of dollars of lost crops annually, nematode infections are a serious problem for human health and for agricultural production. While there are drugs to treat infections, many pockets of parasites have been identified worldwide that are developing immunity to the standard treatment regimen. In this study we describe a strategy using the model organism *C*. *elegans* as a surrogate parasite to identify several new chemical compounds that may offer additional treatments for infection. We demonstrate how to use our platform to identify compounds that are specific in their effect to nematodes and are not simply biocides. We also show through genetic and molecular analysis in this organism that we can quickly identify the mode of action of any new compound. Most critically, we show that a compound first identified in a free-living nematode, *Caenorhabditis elegans*, is also effective on a parasitic nematode, *Meloidogyne hapla*. With this result and considering the level of sequence conservation across much of the nematode phyla we believe our strategy can be more widely applied to find new anthelmintics.

## Introduction

Over 60 species of nematodes parasitize humans. According to a 2005 report by the World Health Organization (WHO), approximately two billion humans have helminth infections worldwide [1], and the problem has worsened in the intervening decade [2]. Parasitic nematodes are the most common infectious agents and produce a global disease burden greater than other conditions such as malaria and tuberculosis [3]. Nematodes also imperil the world’s food supply, as they are infectious parasites of livestock and plants. Plant-parasitic nematodes are recognized as one of the greatest threat to crops throughout the world, destroying over 12% of global food crop production annually and costing an estimated 157 billion US dollars annually [4,5]. Unfortunately, the arsenal of effective compounds is limited. Ivermectin, a nematicide that activates glutamate-gated chloride channels resulting in nerve and muscle hyperpolarization and worm paralysis [6–8] is currently the drug of choice and is recommended by WHO to treat nematode infections. Nematode resistance to anthelmintic drugs in general, and ivermectin in particular, is growing worldwide which poses a problem for both human populations and livestock [9–15]. In some respects this resistance is expected because ivermectin has been used for large-scale public distribution on an annual basis since 1987. Anthelmintic resistance can arise through genetic alteration of the drug target [16,17] or by different strategies that worms use to reduce access of the drug to the target (reviewed in [18]). Recently, mutations in *dyf-7*, a gene known to be involved in anchoring the dendritic tips of sensory neurons [19] has been shown to be associated with resistance to ivermectin/macrocyclic-lactone-related drugs worldwide [20]. These data support an earlier observation that amphid sensory neurons (which communicate with the environment) in ivermectin resistant *Haemonchus contortus* are defective, displaying reduced sensory cilia and general morphological degeneration [21]. It is speculated that the open channels of these sensory neurons offers a ready pathway for absorption of ivermectin, channels that are lost or collapsed in animals with *dyf-7* mutations. These many potential modes of resistance, combined with widespread reports of worldwide anthelmintic resistance (in particular to ivermectin), are cause for concern and have many organizations calling for the development of new drugs to treat nematode infections.

Parasitic nematodes are often difficult to maintain in the laboratory, and the lack of molecular and cellular tools poses experimental challenges to studying the parasites directly in a controlled setting. Fortunately, many anthelmintics have the same biological effect on non-parasitic nematodes as they do on parasitic animals (reviewed in [22,23]). This biological conservation opens opportunities to use non-parasitic nematodes such as *C*. *elegans* for discovering new compounds that act as anthelmintics. In their review on anthelmintic drugs Holden-Dye and Walker (2007) ask, “Is *C*. *elegans* a model parasite?” and while they conclude it is not appropriate for examining the parasite life cycle, it is a very good model for comparative physiology and pharmacology for the phylum Nematoda. This was demonstrated experimentally by the work of Dr. Anthony Stretton’s group in the 1980’s comparing the parasitic nematode *Ascaris suum* to *C*. *elegans* (neural wiring, [24]; acetylcholine in excitatory and GABA inhibitory neurons, [25,26]). These studies plus many more in the intervening years lead us to contend that *C*. *elegans* can be used as an efficient surrogate for discovering small molecule perturbants of infectious nematodes. Comparative genomic studies suggest a great deal of sequence similarity across the phylum Nematoda [27], but also some very notable differences, especially among the filarial group (see *Loa Loa* sequence; [28]). Even considering these differences *C*. *elegans* shares almost 13,000 (~70%) of its genes with other species of nematodes, including many shared genes with the filarial group [27].

Rand and Johnson [29] pointed out that the ease of handling and the tools available for this organism make *C*. *elegans* ideal for drug screening and the discovery and characterization of drug targets. It was they who coined the term ‘genetic pharmacology’. Despite these attributes, there have been surprisingly few published studies that take advantage of this organism for primary drug screening (reviewed in [23,30]) with some notable exceptions (see for example, [31–37]). There are probably a number of factors contributing to why *C*. *elegans* has not been more widely used for drug discovery, but a principal one is that, until recently, there were few reports utilizing high throughput phenotypic screening.

Towards the goal of expanding the scope of *C*. *elegans* for drug screening we have developed a rapid, high throughput screening protocol that permits a wide sampling of the chemical landscape for potential nematicides in a very efficient manner. At a rate of 1,900 compounds per hour, the ease and pace of primary screening of nematode populations using this platform is unprecedented and has led to efficient identification of new lead chemicals to be developed as nematicides. Chemicals that will be effective on nematodes may be rare [38], but by sampling a sufficiently large number of chemicals rapidly, we have a reasonable likelihood of identifying new anthelmintics. Here we report the results from screening of 25,986 compounds from the Maybridge and Chembridge chemical libraries. We have identified several hundred nematode-active compounds, and we focus on 14 compounds with biological effects on growth and fecundity. We also demonstrate that nematodes are more sensitive to several of these compounds compared to several other organisms. This suggests to us that it is possible to use this strategy to develop novel classes of compounds specific to anthelmintic, which should lower the overall environmental toxicity of these compounds. This should facilitate their use in multiple conditions, settings and locations thus making it possible to decrease the overall parasitic nematode burden in both humans and livestock.

## Materials and Methods

### *C*. *elegans* strains and culture

*C*. *elegans* strains were maintained using standard techniques as previously described [39].The following *C*. *elegans* strains were used: VC2010, the Moerman lab subculture of the Bristol isolate of *C*. *elegans* (N2; [40]) for compound screening; DM7448 (VC20019 carrying *gkEx1*, an extrachromosomal array that confers body-wall muscle YFP), DR103 *(dpy-10(e128) unc-4(e120))*, DR1705 (*lin-31(u301) dpy-10(e128)*), and DR1489 (*dpy-17(e164) unc-36(e251)*) for resistance mutation mapping; and CB3474 (*ben-1(e1880)*, benomyl resistant [41]), RB2119 (*acr-23 (ok2804)*, monepantel-resistant [31]), CB193 (*unc-29*,*(e193)* levamisole resistant [42]), and DA1316 (*avr-14(ad1302); avr-15(vu227); glc-1(pk54)*, ivermectin resistant [7,8]) for testing known drug resistance pathways. We also used many strains from the *C*. *elegans* Million Mutation Project (MMP [43] and http://genome.sfu.ca/mmp/search.html), or the *C*. *elegans* Gene Knockout Project [44], in order to test *pink-1* and *mev-1* mutations for drug resistance and to explore modes of action. For *pink-1* we used strains RB2547, VC20205, VC20423, VC20470, VC20521, VC20546, VC20588, VC30182, VC40096, VC40194, VC40287, VC40373, VC40385, VC40392, VC40489, VC40527, VC40694, VC40738, VC41008 and VC30104. For *mev-1* we used VC20501, VC20602, VC40781, VC40799, VC40934, VC20417, VC30090, VC30107, VC40073, VC40304, VC40350, VC40391, VC40533, VC40576, VC40631, VC40764, VC40770, VC41025, VC20401, VC20587, VC40186, VC40193, VC40364, VC40423, VC40752, VC40765, VC20295, VC30120, VC40386, VC40570 and VC40903.

### Source of compound libraries and procedure for anthelmintic primary and secondary screens

The following compound libraries were used: 3,584 compounds from the Canadian Chemical Biology Network library (Prestwick, Sigma LOPAC, Microsource Spectrum and BioMol collections) including FDA-approved drugs; 16,000 compounds from the Hitfinder library (Maybridge); and 10,000 compounds from the DIVERSet library (ChemBridge).

All compound libraries were stored in 96-well plates, with 80 drugs per plate (columns 1 and 12 left empty). A BioGrid BG600 pinning robot (Digilab / BioRobotics) was used for compound transfer. The robot uses a 96-pin tool (0.7 mm dia.) to transfer a nominal volume of 340 nL of compound. For retesting, compounds were hand pinned from the original library plates to assay plates using a single 0.7 mm dia. pin. The assay plates were 96-well clear flat-bottom microplates containing 2% χ1666 *E*.*coli* bacteria in 0.5X Liquid Nematode Growth Media (LNGM) plus cholesterol (Bacteria were mass-grown in rich media, and harvested by centrifugation to produce a thick paste of bacterial cells that was kept frozen at -20**°**C. Cells were resuspended at 2% W/V in 0.5X LNGM to produce liquid worm food). Using a COPAS Biosort (Union Biometrica), two L4 to young adult stage VC2010 worms were added to each well of the plate in a final volume of 45 μL. After five days of exposure to the compounds, the plates were scanned and the data analyzed. A five day time period was chosen to be able to observe effects over all stages of the life cycle and through at least one round of replication. The initial proof-of-principle primary screen was performed manually using a dissecting microscope. Phenotypes scored included decreased motility, reduced brood size, and/or death as described previously [45]. For high throughput screening of the Chembridge and Maybridge compound libraries we used the program WormScan [46], which was adapted for screening 96-well flat-bottom LNGM plates. Using WormScan it was possible to screen four 96-well plates at a time. Plates were scanned twice, at a resolution of 1200 dots per inch, 16-bit grayscale, producing a jpeg image. The light intensity produced by the scanner was sufficient to cause negative phototaxis equivalent to a physical stimulus for mortality determination. The time required to scan four 96-well plates using an Epson Perfection V700 Photo Scanner was less than ten minutes. The time to screen a single plate was less than 1.2 minutes, with less than ten seconds between scans.

**Fig 1:**
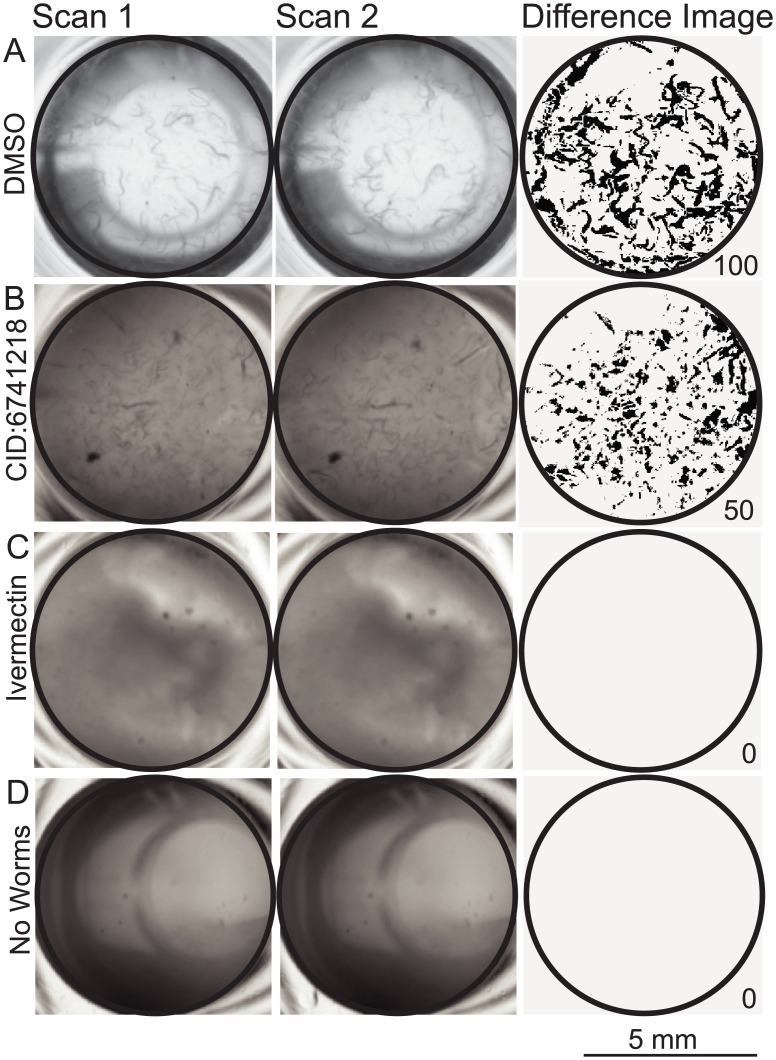
WormScan Score Analysis. (A) Two VC2010 L4 stage *C*. *elegans* in a 1% DMSO control well that contains no drug, two sequential scans are taken after five days of exposure, the difference image generated for the region of interest (black circle) gives a WormScan Score of 100. (B) Two VC2010 L4 *C*. *elegans* exposed to 43 μM of CID 6741218 for five days exposure resulted in reduced brood size, reduced behavioral response to light stimulus and increased mortality, giving a WormScan Score of 50. (C) Two VC2010 L4 stage *C*. *elegans* exposed to 50 μM of Ivermectin for five days of exposure, giving a WormScan Score of 0, which resulted in mortality. (D) A well that contained no *C*. *elegans* gives a WormScan Score of 0. The scale bar applies to all images.

WormScan was used to screen for the same phenotypes as in the manual screen (reduced brood size, reduced behavior/motility response and increased mortality). Two sequential scans for each 96-well plate were aligned to a reference region of interest (ROI) [47]. Image analysis was based on a difference image score calculated for each ROI. The difference image score (WormScan score) was normalized using a “percent of control” method to derive a normalized WormScan score [48]. Fig 1 shows original scans, the difference image and normalized WormScan score for several controls and one of the positive test compounds. Wild-type growth in 1% DMSO (Fig 1A) results in a normalized WormScan score of 100. Exposure to 50 nM ivermectin gives a score of 0 (Fig 1C), and complete absence of worms gives a score of 0 (Fig 1D). For the primary screen the WormScan score was calculated using custom macro scripts written for Fiji [49]. For secondary screening and any subsequent studies the WormScan score was calculated using a standalone Java program (See supplemental). Probit regression of mortality was calculated using Mathematica 8.0.

### Phylogenetic testing of putative anthelmintics

Potential anthelmintic compounds identified from primary screening were further tested against mammalian cells and yeast to determine whether they had deleterious effects in those organisms. Human embryonic kidney (HEK293) cells were grown in Dulbecco’s Modified Eagle’s Medium (DMEM; Sigma) containing 5% fetal bovine serum (FBS; Sigma) in 96-well plates tissue culture plates. Compounds were added 24h post-plating at 10, 30, 100, or 300 μM final concentrations. Dimethyl sulfoxide (DMSO) was used as a negative control at 0.1%. Cells were treated for 24 or 48 hours, after which time viability was assessed using a 3-(4,5-dimethylthiazol-2-yl)-2,5-diphenyltetrazolium bromide (MTT) colorimetric assay [50]. Percent viability was determined by comparing treatment values to DMSO control values.

The *S*. *cerevisiae* yeast strain BY4743 (*MAT*a/α *his3*Δ*1/his3*Δ*1 leu2*Δ*0/leu2*Δ*0 LYS2/lys2*Δ*0 met15*Δ*0/MET15 ura3*Δ*0/ura3*Δ*0*) [51] was used for all yeast experiments and was grown using yeast extract peptone dextrose (YPD) media at pH 7 [52]. BY4743 was grown overnight to initial log phase and was then grown with the compound identified in the *C*. *elegans* assay. The growth rate was quantified by continuously monitoring the optical density of the liquid yeast growth at OD_600_. The compound was screened in a dose-titration in YPD as described [53]. The IC_50_ was the concentration required to reduce the growth rate or Average G value by 50% compared to a vehicle control (2% DMSO).

### Anthelmintic testing on other species of nematodes

A laboratory population of the plant-parasitic northern root-knot nematode, *Meloidogyne hapla*, was initiated by inoculating a tomato seedling growing in a 500 ml pot of 50:50 pasteurized sandy soil:peat with ~100 infective second-stage juveniles (J2) extracted from infested soil from a vineyard in the Okanagan Valley. The population was thereafter reared on tomato under greenhouse conditions. Prior to experimentation, galled roots were removed from heavily infested tomato plants, washed over a sieve with tap water, and then placed on Baermann funnels. J2 hatchings over the first three days were collected and suspended in tap water at a concentration of 100 J2/ml. Replicate 24-well cell culture plates were seeded with 0.2 ml of J2 suspension. Dilutions of CID 2747322 in distilled water were then added to the wells to result in four replicate wells of each of six concentrations in each plate. Concentrations tested spanned 0 to 320 μM and 0 to 160 μM in the two separate runs of the experiment. Nematodes were observed using an inverted microscope at 40X. On each observation date, a transect through each well was observed and J2 within the transect were scored as mobile or immobile. J2 that had taken on a straight posture were scored as immobile. Recently hatched J2 of *M*. *hapla* normally move continuously but slowly and always have a curved posture. The integrity of the esophageal-intestinal junction, which disintegrates in dead J2 [54], was observed to eventually disintegrate in J2 immobilized by CID 2747322.

### Selection and identification of mutations conferring resistance to compound CID 2747322

A standard ethyl methanesulfonate (EMS) mutagenesis protocol [55] was carried out using VC2010 in order to generate mutants resistant to CID 2747322 exposure. First larval stage (L1) F2 progeny of mutagenized P0s were exposed to CID 2747322 at 200 μM in liquid worm food in 24-well tissue culture plates for five days at room temperature, and two independently isolated resistant lines (VC3614 and VC3615) were identified. Resistant animals were identified as well moving viable animals. Each of these was outcrossed three or four times with DM7448 (VC20019 strain with body-wall YFP marker), selecting after each outcross one animal homozygous for a mutation conferring resistance to the compound. The purpose of the crossing was to reduce unrelated EMS-generated mutations (S1 Fig for details [56]). For each of the two independent mutations, the homozygous mutant line resulting from the final outcross (VC3635 and VC3631, respectively) was kept for further analysis. Genomic DNAs from VC3635 and VC3631 were extracted and sequenced, and candidate mutations were identified [40]. Standard three-factor genetic mapping was done for each of the putative candidate regions. Two sets of crosses on chromosome II used DR103 (*dpy-10(e128) unc-4(e120)*) and DR1705 (*lin-31(n301) dpy-10(e128)*), and another set of crosses on chromosome III used DR1489 (*dpy-17(e164) unc-36(e251)*) to identify the genes conferring resistance.

### Determining mitochondrial copy number variation in wild type and *pink-1* strains

Sequence reads from the MMP [43] were obtained from the Short Read Archive and realigned on the reference *C*. *elegans* genome using the BWA aligner. The mtDNA copy number was then estimated simply by scaling the number of reads aligning per base of mtDNA to the corresponding value for the autosomes and multiplying for a factor of two to account for diploidy.

### Structure Activity Relationship (SAR) and cheminformatics

A 3D similarity score was generated with the chemistry informatics tool Screen3D [57] using the atom matching algorithm and allowing the query and template molecules to be flexible. The compounds were categorized into similar groups using the Library MCS hierarchical clustering program Chemaxon (Chemaxon: Budapest H, Library MCS, version 6.0.1,2013). Singletons (molecules which did not belong to any cluster) were removed and all the remaining scaffolds were saved.

### Homology modeling for determining drug specificity

Protein homology models were generated for the four protein subunits of mitochondrial complex II for *C*. *elegans*, SDHA-1, SDHB-1, SDHC-1 (MEV-1) and SDHD-1 using Modeller version 9.15 [58]. Primary sequence alignment of the template with *Ascaris suum* sequence was generated with ClustalW version 2.0.12 [59]. Screen3D was used to model the binding of CID 2747322 to the crystal structure of mitochondrial complex II. Structural models and docked ligands were visualized with pymol version1.7.4 [60].

## Results

### Screening libraries

As a proof-of-principle experiment, we first carried out a manual phenotyping pilot study with a set of 3,584 compounds that includes many approved drugs and we identified over 95% of the known nematicides present in the collection, including ivermectin and other macrocyclic lactones, organophosphates, lisurides, emodepsides, benzimidazoles and their derivatives, and amine acetonitrile derivatives (AADs). Most of these compounds are actually biocides, with the exception of AADs. This pilot study, done in a blinded manner (with the compound identity unknown to the scorer), gave us the confidence that the screen is effective and efficient at identifying anthelmintic compounds (S1 Table).

**Fig 2:**
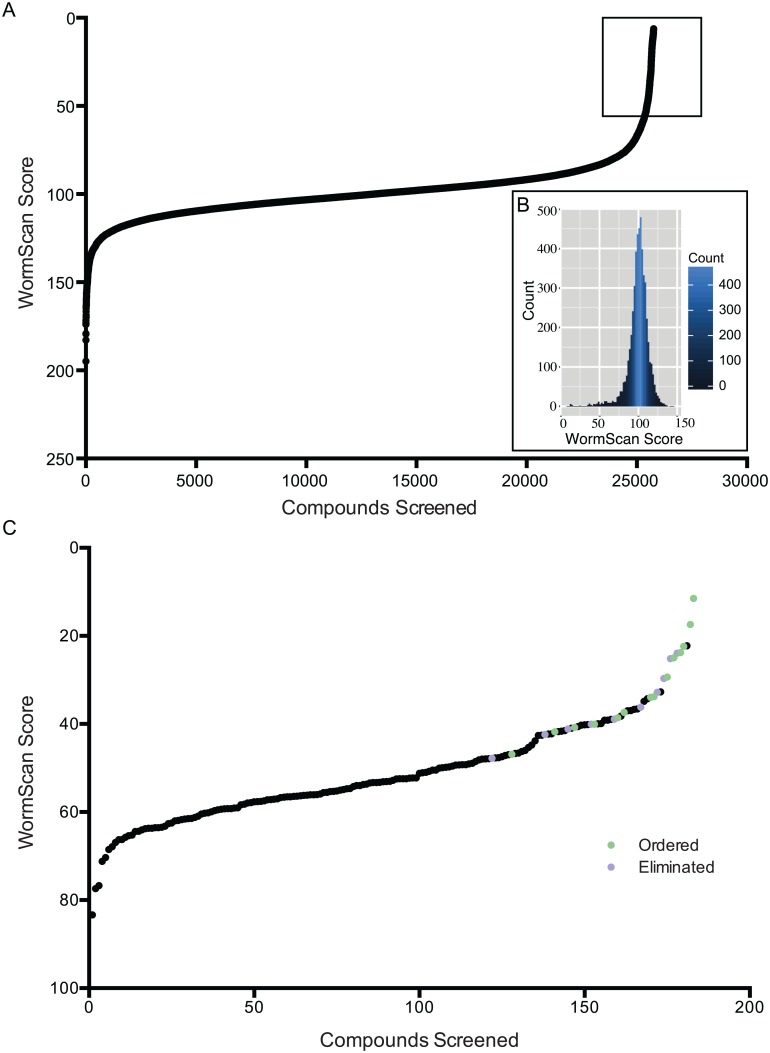
WormScan Scores from initial compound screen and confirmation of hits. (A) Two VC2010 L4 *C*. *elegans* were sorted into each well of a 96-well flat-bottom plate. *C*. *elegans* were exposed to 43 μM for each of the 26,000 compounds from the Maybridge and Chembridge libraries for 5 days and then screened and sorted by WormScan Score, which was normalized by percent of control wells. The 404 top anthelmintic candidates from the initial screen are highlighted by the black box. (B) The WormScan Scores of the 5,152 controls from the compound screen are shown in histogram with a bin width of 2. The mean WormScan Score for the controls is 100. (C) The top 404 compounds were re-pinned for two more biological replicates and displayed here are the top 184 active compounds. After a series of filters were applied to these 184 compounds, 14 compound candidates were retained for further testing.

We next screened 26,000 compounds from the ChemBridge DIVERset and Maybridge Hitfinder compound libraries. The initial 3,200 compounds (40 plates) from these libraries were screened using the automated WormScan protocol and manually with 100% agreement for the top 37 hits. This gave us the confidence to do further screening using only WormScan. We normalized the raw WormScan scores to correct for plate-to-plate variability (Fig 2a and 2b) to arrive at a ranked list of nematode-active agents after 5 days of compound exposure. Whereas previous *C*. *elegans* high throughput drug screens were limited to measuring motility [61], we are able to quantify a larger range of phenotypes (for example, Fig 1b). We focused on the easily identified phenotypes of lethality, reduced fecundity, slow growth, and immobility. Besides their ease of scoring, these particular phenotypes report relevant characteristics we wish to observe in relation to any potential anthelmintic. Retesting active compounds from the primary screen two additional times resulted in the identification of 137 chemicals affecting nematode survival, fecundity or behavior (Fig 2c). We collapsed these 137 compounds to 14 (see Table 1), using filters related to strength of phenotypic effect, public bioassay data and any known general toxicity and promiscuity of the compounds. To avoid “rediscovering” known drugs, we gave lower priority to compounds that had a described mechanistic activity or whose chemical structure was similar to established anthelmintics [62,63]. We reasoned that emphasizing novel chemical backbone structures might lead to identifying biochemical pathways in the nematode that have not yet been exploited [64]. Even so some known chemical backbones did come through our filters (see below). In patent searches using the 14 selected compounds as queries, we found that Bayer AG recently patented one compound for use on intestinal parasites [65]. This patented compound (1–36) has structural similarity to our best hit, N-[2-(4-methoxyphenoxy)ethyl]-2-(trifluoromethyl)benzamide (CID 2747322), with a 3D Tanimoto similarity score of 0.82 to our compound. This independent discovery can be viewed as validation of our screening approach for identifying new anthelmintic drugs. It does emphasize however, that our filters for unique chemical backbones are not full proof as they did not exclude some previously identified anthelmintics (specifically note fluopyram in a later section).

**Table 1:** Phylogenetic testing to identify nematode specific compounds. The compounds identified from the *C*. *elegans* screen are tested against yeast (BY4743) and mammalian cells (HEK293) to determine the IC_50_ values. Also tested are three commonly used nematicides, fluopyram, ivermectin and benomyl.

Drug	Nematode IC_50_ (μM)	Mammalian IC_50_ (μM)	Yeast IC_50_ (μM)
D1 CID:3254982	17	37	140
D2 CID:2238042	357	67	338
D4 CID:796072	7	28	> 1000
D5 CID:6456299	> 500	184	> 1000
D6 CID:766260	282	44	113
D7 CID:2222671	> 500	234	> 1000
D8 CID:6741218	8	97	> 1000
D10 CID:6745334	32	30	437
D13 CID:2743002	> 500	54	20
D15 CID:2823320	40	45	> 1000
D16 CID:2740991	19	83	> 1000
D17 CID:2745791	99	54	> 1000
D18 CID:2747279	13	190	> 1000
D19 CID:2747322	18	171	> 1000
Fluopyram	0.05	300	> 1000
Benomyl	60	8.0	750
Ivermectin	0.03	300	> 1000

To do structural clustering of the fourteen compounds we followed the chemistry informatics protocol used by the Burns study [35], which is a standard way of grouping compounds. Using the procedure two molecules are defined as being in a similar class if they have a Tanimoto score of greater than 0.55 (pairwise Tanimoto/FP2 score <0.55). A network of relatedness is established using a pairwise comparison of each hit to all hits (both from this study and Burns et al [35]). S2 Table displays the 2-D structure for each of our 14 compounds plus the Tanimoto score for each compound. Five of our compounds, including three of our best hits, CID 2747322, CID 2747279, and CID 6741218, fall into clusters defined in Burns et al [35], four compounds, including CID 796072, fall into orphan clusters identified by Burns et al [35], and five appear to be new chemistry not previously identified to have any affect on nematodes. Note that similarity in structural class does not necessarily imply similarity in function or in mode of action. For example, CID:6741218 with a Tanimoto score of 0.93 is highly similar to the compound WACT162 identified in the Burns et al study [35], but while CID:6741218 is highly toxic to nematodes, WACT162 only reduces growth.

### Identifying nematode-specific compounds by phylogenetic testing

From the perspective of identifying potential anthelmintics, we are primarily interested in compounds whose activity is restricted to nematodes. Each of the 14 compounds identified was tested across different phylogenetic groups (including yeast, mammalian cell lines and other nematode species). An order of magnitude difference in toxicity between mammalian cells and nematodes is ideal [66]. However, *C*. *elegans* have been shown to be more resistant towards established anthelmintics compounds when compared to parasitic nematodes [67]. To date we have identified five compounds that have a high “nematode index”, which we define as having a significantly more potent effect on nematodes than on other organisms. Two of these "nematode actives" are perhaps marginal as CID 796072 and CID 6741218 only have a four-fold greater sensitivity in *C*. *elegans*. The other three, CID 2740991, CID 2747279 and CID 2747322, are more convincing with each having a 10-fold or greater sensitivity (see Table 1).

We wished to compare the relative effectiveness of our new compounds to known anthelmintics in this assay. For this purpose we chose ivermectin, benomyl and fluopyram. Floupyram is a Bayer produced fungicide that has recently been approved as an anthelmintic as well [35]. Not unexpectedly benomyl shows no special sensitivity in nematodes over mammalian tissue cells. In contrast ivermectin and fluopyram show a greatly increased effectiveness in nematodes over the mammalian tissue culture cells or yeast (Table 1). They are also effective on nematodes at a much lower concentration than any of our newly identified compounds. From our chemical relatedness analysis our compounds CID:2747279 and CID:2747322 are in the same chemical class as fluopyram and the Burns et al compound WACT 11 [35].

### CID 796072, CID 6741218 and CID 2747322 do not act through the benzimidazole, levamisole, ivermectin or AAD biochemical pathways

An advantage of using *C*. *elegans* to characterize new potential anthelmintics is that we can use the extensive genetic toolkit for this organism to explore the mode of action of prospective anthelmintics. For example, to better understand the potential development of resistance to our newly-identified compounds, we tested three of the new compounds we discovered, CID 796072, CID 6741218 and CID 2747322 on strains that are resistant to the common anthelmintics benzimidazole, levamisole, ivermectin and AAD (Table 2). If these resistant mutant lines are sensitive to a compound at doses that kill wild type animals we can deduce that the compound acts through a different pathway and perhaps has a novel target and mechanism of action [68]. The known anthelmintic-resistant lines provide both positive and negative controls (e.g. the ivermectin resistant worm should be resistant to ivermectin in our assay but not to benomyl, and vice versa). Encouragingly, examination of Table 2 reveals that CID 796072, CID 6741218 and CID 2747322 are equally effective on the four resistant lines as on the wild type strain VC2010 and at similar concentrations. While we felt these results lent credence to the idea that we have indeed identified novel-acting anthelmintics further studies (see below) suggests that at least CID 2747322 acts through a similar biochemical pathway to fluopyram.

**Table 2:** Mapping compound mode of action using *C*. *elegans* anthelmintic resistant strains. The IC_50_ of different *C*. *elegans* anthelmintic resistant strains after 5 days exposure, 2 L4 stage *C*. *elegans* were placed in a 96-well for three biological replicates.

Strain (resistant to)	Ivermectin (μM)	Benzimidazole (μM)	Levamisole (μM)	D4 CID 796072 (μM)	D8 CID 6741218 (μM)	D19 CID 2747322 (μM)
VC2010 (wild type)	0.03	61	4	7	8	18
DA1316 (Ivermectin)	7	59	8	8	3	16
CB3474 (Benzimidazole)	0.01	> 1000	2	2	10	4
CB193 (Levamisole)	0.01	113	> 250	3	4	14
RB2119 (amino-acetonitriles)	0.01	28	4	2	6	5
VC3635 (D19)	0.01	118	6	9	12	> 500
VC3631 (D19)	0.01	91	10	11	13	> 500

### The effect of CID 2747322 on *C*. *elegans*, *C*. *briggsae* and *M*. *hapla* growth and survival

WormScan is a powerful tool to identify compounds with potential effects on the nematode, but it is still necessary to do manual inspection of wells with few animals to determine details of growth, behavior and fecundity. Wild-type worms grown in 90 μM CID 2747322 have brood sizes reduced by 97%. These small broods of live animals are sickly and uncoordinated and arrest at the first larval stage (L1) and do not develop further while maintained in drug (See S2 Fig). All current classes of anthelmintic have greater specificity towards certain life-stages [69]. It is interesting that when removed from the drug L1s will recover and develop, and generally become fertile adults (See S3 Fig). This suggests a reversible inhibition by CID 2747322, which we do not observe with fluopyram. For fluopyram animals are killed.

It is important to note that while such specificities of effect on *C*. *elegans* are desirable, the ultimate test requires direct testing on other nematodes, specifically parasitic nematodes. To this end, we have tested two species and both are sensitive to CID 2747322. We first examined to see if other free-living nematodes are sensitive to the drug and found that *Caenorhabditis briggsae* displays a sensitivity range similar to *C*. *elegans*. The IC_50_ for *C*. *briggsae* is 16 μM, while for *C*. *elegans* the IC_50_ is 18 μM (S4 Fig). More critical was our test of effects on infective J2 of the plant parasitic nematode, *Meloidogynae hapla*. Immobilization was observed in the 160 and 320 μM concentrations at 24 hours after exposure initiation, and increased through day 10. For the 10 day exposure (S5 Fig), the IC_50_ was calculated to be 129 μM or 44 μg/ml. Plant parasitic nematodes have found to be generally more resistant to anthelmintics compared to *C*. *elegans* [70]. While the *M*. *hapla* J2 appeared to be considerably more tolerant to the compound than *C*. *elegans*, it was comparable to the sensitivity of *M*. *incognita* J2 to avermectin [71]. Unlike *C*. *elegans*, which can recover after a several day exposure to the compound, *M*. *hapla* die after extended exposure to the compound.

### Delineating the mode of action of CID 2747322

To aid in identifying affected pathways we selected for animals resistant to CID 2747322 after EMS mutagenesis. Resistant lines were outcrossed and subjected to genetic mapping and Sanger sequencing to identify the responsible genes and mutations. This forward genetics approach is a validated and time-honored means to identify genes involved in drug resistance [7,8,31,34]. Because of the unbiased nature of this approach, it can identify direct targets of the compound and/or the pathway affected by the compound as well as additional modes of resistance, such as drug uptake, export, or sequestration (see for example *dyf-7*, [20]). Screening the F2 progeny of 150 P0 animals subjected to mutagenesis resulted in two independently isolated CID 2747322 resistant lines, VC3631 and VC3635. These two lines confer similar levels of recessive drug resistance. We used a variation on the outcrossing and Whole Genome Sequencing (WGS) strategy described by Zuryn and Jarriault ([56]; see S1 Fig for details) coupled to standard genetic three-factor mapping to identify the two genes responsible for the observed resistance.

### Mutations in *pink-1* confer resistance to CID 2747322

Analysis of the whole genome sequencing (WGS) data for VC3635 identified 23 unique Single Nucleotide Variants (SNVs) spread along the length of chromosome II, and presumably one of these SNVs is responsible for the observed drug resistance. Three-factor mapping using *dpy-10 unc-4* yielded 15 Dpy recombinants that were all drug sensitive and eight Unc recombinants that were all drug resistant. Further three-factor mapping with *lin-31 dpy-10* yielded 20 Lin recombinants, six of which were resistant to the drug. While there are nine SNVs within coding regions in the mapped interval, we focused our attention on three SNV-containing genes, *dsh-1*, *pho-1* and *pink-1*. We sequenced the six drug-resistant Lin recombinants and found that all six carried a wild type allele at the *dsh-1* locus and one of five carried a wild type allele at the *pho-1* locus. Only the *pink-1* locus carried the mutant allele in all the drug-resistant recombinant lines, strongly implicating *pink-1* as the gene conferring resistance. We also undertook a reverse genetic approach, testing 20 strains from the Million Mutation Project (MMP) collection that harbor *pink-1* mutations and found that six strains with *pink-1* missense mutations are resistant to the drug (Table 3). We conclude that *pink-1* (EEED8.9) is the gene in strain VC3635 responsible for resistance to CID 2747322. The *pink-1* gene encodes a predicted serine/threonine kinase that is most similar to the *Drosophila* and human PINK1 (PTEN-induced kinase-1) protein kinases.

**Table 3:** Multiple alleles of PINK-1 confer resistance to CID 2747322. To confirm that *pink-1* (*gk3615*) is the cause of resistance in strain VC3635 strain to CID 2747322 we tested several *pink-1* mutant lines from *C*. *elegans* million mutation project (MMP). There are 6 MMP strains that are resistant to CID 2747322 exposure, 12 strains that show wild-type (none) resistance and two strains that are sensitive towards exposure.

Strain	Effect	Resistance
VC20205	W562stop	none
VC20423	V373I	none
VC20470	E162K	none
VC20521	P226S	sensitive
VC20546	P32L	sensitive
VC20588	A628T	none
VC30182	G415E	none
VC40096	M1I	none
VC40194	D378N	resistant
VC40287	S341N	resistant
VC40373	A365T	none
VC40385	L232F	resistant
VC40392	T65I	none
VC40489	A628T	resistant
VC40527	V334I	none
VC40694	M556L	resistant
VC40738	E353K	none
VC41008	L180F	resistant
VC30104	Knockout	none
RB2547	Knockout	none

PINK-1 is an important protein involved in mitochondrial homeostasis. Because it is part of the pathway to remove damaged mitochondria by autophagy (reviewed in [72]) we considered if mitochondrial DNA (mtDNA) copy number may be altered in these mutants [73]. Reanalysis of existing WGS data for the MMP strains allowed us to examine the mtDNA copy number in six MMP *pink-1* mutant strains, three that are sensitive to the drug, and three that are resistant. We compared the ratio of mtDNA copy number to that of the genome copy number and found no correlation between resistance and mtDNA copy number (Table 4). The largest ratio of mtDNA to genomic DNA is actually in a sensitive strain, but the ratios for all six are remarkably similar. Simple amplification of mitochondrial DNA copy number is therefore not the explanation for *pink-1* resistance.

**Table 4:** Copy number mtDNA of PINK-1 MMP strains. The MMP strains were previously sequenced to a coverage of ≥15x. The relative mtDNA copy number was calculated by assuming that the chromosomes have two copies it is possible to scale the number of reads with the size of the chromosome and mtDNA and get an estimate of the copy number of mtDNA.

Strains	Relative mtDNA copy number
VC20588	70
VC30182	101
VC40194	59
VC40287	87
VC40385	56
VC40527	50

### Mutations in *mev-1* confer resistance to CID 2747322

Analysis of the WGS data for VC3631 identified nine unique SNVs clustered in the central region of chromosome III. Three-factor mapping using *dpy-17 unc-36* yielded 15 Dpy recombinants, all resistant to 250 μM CID 2747322, and seven Unc recombinants all sensitive to the drug. This suggested that the mutation conferring resistance lies to the right of *unc-36*, a region containing *mev-1* and Y39A1A.9. At this point we took a candidate gene approach, as *mev-1* had recently been implicated in resistance to another potential anthelmintic [34]. We sequenced six drug-resistant Dpy recombinant lines and three non-resistant Unc recombinant lines for the *mev-1* locus. All the drug-resistant recombinant lines have the *mev-1* associated SNV and all the drug-sensitive Unc lines contain wild-type versions of *mev-1*. We also examined five strains from the MMP collection harboring *mev-1* missense mutations, and two of these, VC20602 and VC40781, showed resistance at the same level as VC3631 (Table 5). We conclude that *mev-1* (T07C4.7) is the gene in VC3631 responsible for resistance to CID 2747322. The *mev-1* gene encodes the *C*. *elegans* ortholog of the succinate dehydrogenase cytochrome b560 subunit, an integral membrane protein that is a subunit of mitochondrial respiratory chain complex II (ubiquinol-cytochrome c reductase).

**Table 5:** Multiple mitochondrial complex II mutations confer resistance to CID 2747322. To confirm that mev-1 (*gk361*) is the cause of resistance in strain VC3631 to CID 2747322 we tested mitochondrial complex II mutant lines from the *C*. *elegans* Million Mutation Project (MMP). It was found that 7 of the MMP strains for the mitochondrial complex II were resistant towards CID 2747322 exposure, 15 of the strains had wild-type (none) resistance and 9 strains were sensitive towards exposure.

Strain	Gene	Chromosome	Effect	Resistance
VC20501	*mev-1*	III	S178N	none
VC20602	*mev-1*	III	G77S	resistant
VC40781	*mev-1*	III	Y152D	resistant
VC40799	*mev-1*	III	R52C	none
VC40934	*mev-1*	III	G88E	none
VC20417	*sdha-1*	X	E305K	none
VC30090	*sdha-1*	X	G363E	sensitive
VC30107	*sdha-1*	X	S292T	sensitive
VC40073	*sdha-1*	X	S142F	sensitive
VC40304	*sdha-1*	X	P385S	none
VC40350	*sdha-1*	X	V131I	none
VC40391	*sdha-1*	X	G335E	resistant
VC40533	*sdha-1*	X	R482C	resistant
VC40576	*sdha-1*	X	D317N	sensitive
VC40631	*sdha-1*	X	H504Y	none
VC40764	*sdha-1*	X	A425V	sensitive
VC40770	*sdha-1*	X	E472K	none
VC41025	*sdha-1*	X	S570F	sensitive
VC20401	*sdhb-1*	II	S209L	resistant
VC20587	*sdhb-1*	II	E294K	none
VC40186	*sdhb-1*	II	D192E	none
VC40193	*sdhb-1*	II	T285I	resistant
VC40364	*sdhb-1*	II	N118I	sensitive
VC40423	*sdhb-1*	II	I280S	sensitive
VC40752	*sdhb-1*	II	A34V	sensitive
VC40765	*sdhb-1*	II	L197F	none
VC20295	*sdhd-1*	II	A30V	none
VC30120	*sdhd-1*	II	L59F	none
VC40386	*sdhd-1*	II	A97V	resistant
VC40570	*sdhd-1*	II	L120F	none
VC40903	*sdhd-1*	II	D36N	none

### Mutations in *pink-1* and *mev-1* do not lead to general drug resistance

The newly identified mutations in these two genes are missense mutations; *gk3613* in MEV-1 is a T66I change and *gk3615* in PINK-1 is a G172E change. Null alleles for *pink-1* do not confer resistance suggesting that altering the protein (i.e. a hypermorphic or neomorphic allele) is required to confer resistance. Null mutations in *mev-1* lead to lethality so no null alleles were tested. To determine if the mutations in *mev-1* or *pink-1* acted via an indirect mode of resistance (e.g. similar to *dyf-7*), we exposed VC3631 and VC3635 animals to the three anthelmintics ivermectin, benzimidazole and levamisole (Table 2). In all three cases the strains displayed the same sensitivity to these drugs, as does the parental wild type strain, VC2010, which leads us to conclude these mutations do not confer general drug resistance.

### The succinate dehydrogenase protein complex and resistance to CID 2747322

**Fig 3:**
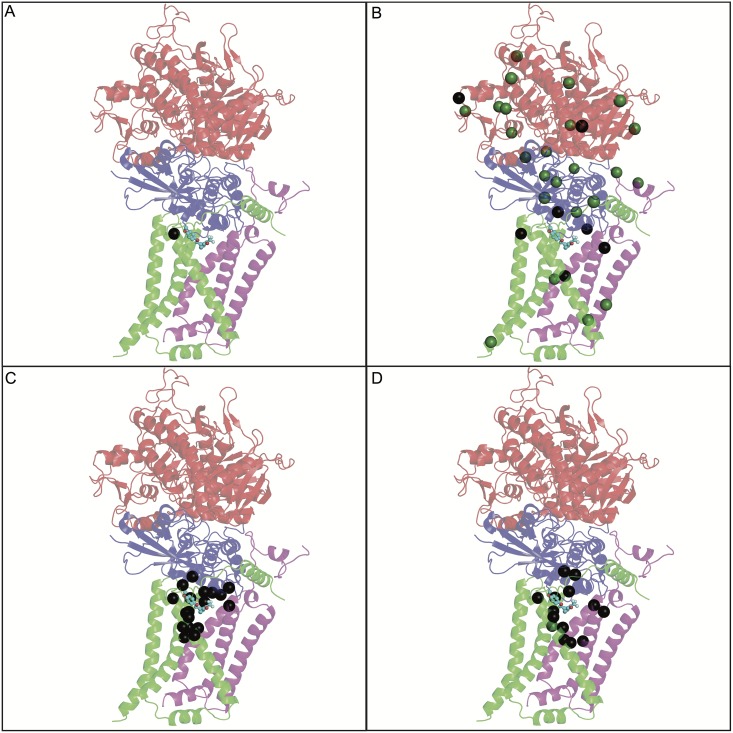
Homology model of *C*. *elegans* complex II with associated amino acid changes conferring resistance to various anthelmintics. In all four panels the complex II subunits are illustrated as follows, SDHA-1 (red), SDHB-1 (blue) or SDHC-1 (*mev-1*; green) and SDHD-1 (purple). The CID 2747322 molecule is colored in cyan and represented as ball and sticks. The chemistry informatics tool Screen3D version 2015 [57] is used to bind CID 2747322 to the crystal structure of complex II with a bound flutolanil analogue. Structural models and docked ligands visualized with pymol [60]. (A) The black sphere of the alpha carbon, T66 position of SDHC-1 (*mev-1)*, is the amino acid alteration in the CID 2747322 resistant strain VC3631. (B) The spheres indicate the million mutation project strains mutations in complex II. Amino acid alterations found to confer resistance towards CID 2747322 exposure are colored in black and wild-type toxicity levels are colored in green. (C) Colored in black are the residues of the pathogenic fungus *Mycosphaerella graminicola*, which give resistance to complex II inhibitors [75]. These positions have been aligned to the *C*. *elegans* position. (D) The black spheres of the alpha carbon residues induce resistance towards exposure from the WACT-11 compound family [34].

CID 2747322 shares some structural similarity to the WACT-11 compound identified by [34]. That study also found that mutations in *mev-1* confer resistance to the potential anthelmintic drug WACT-11, and indeed one of their identified mutations is identical to *gk3613*. Interestingly, they found that mutations in the other three members of the succinate dehydrogenase protein complex also confer resistance. This led us to examine MMP strains with mutations in the other three mitochondrial complex II subunits; *sdha-1*, *sdhb-1* and *sdhd-1*, to see if any of these strains are also resistant to CID 2747322. For thirteen strains with *sdha-1* missense mutations, three are resistant. For eight strains with *sdhb-1* missense mutations, two are resistant. For *sdhd-1*, one of five MMP strains is resistant (Table 5). To better understand how these different alleles might alter drug effectiveness, we mapped all of these mutations plus *gk3613* onto an X-ray resolved protein crystal structure of the mitochondrial complex II of *Ascaris suum* (PDB: 3VRB) [74] (Fig 3a).

The five MMP mutations that confer resistance to CID 2747322 are highlighted, as are the nucleotide positions that, when mutated, do not confer resistance (Fig 3b). The *gk3613* T66I mutation that confers resistance to CID 2747322 was found in the Burns study to confer resistance to the WACT-11 compound series [34] (note position of other WACT-11 resistance sites). Consistent with these observations, we note that resistance mutations for complex II inhibitors found in the wheat pathogen *Mycosphaerella graminicola* [75] have the homologous succinate dehydrogenase position highlighted on the *C*. *elegans* structural model (Fig 3c). It appears that the resistance mutation positions found for CID 2747322 resistance in VC3631, WACT-11 compound family resistance mutations (Fig 3d) [34] and *Mycosphaerella graminicola* all cluster around a putative compound binding pocket. These mutations may change the binding pocket structure and prohibit the inhibitor from binding while still retaining the biological function of the quinone pocket [75,76] (see below for modeling of CID2747322 in the quinone pocket). Our screening of the MMP strains reveals there are additional mutations outside the binding pocket that can also confer resistance. Such “allosteric” resistance mutations that lie outside of the binding pocket for *Sclerotinia sclerotiorum* are also described [77].

### A structure-activity relationship (SAR) for CID 2747322

**Fig 4:**
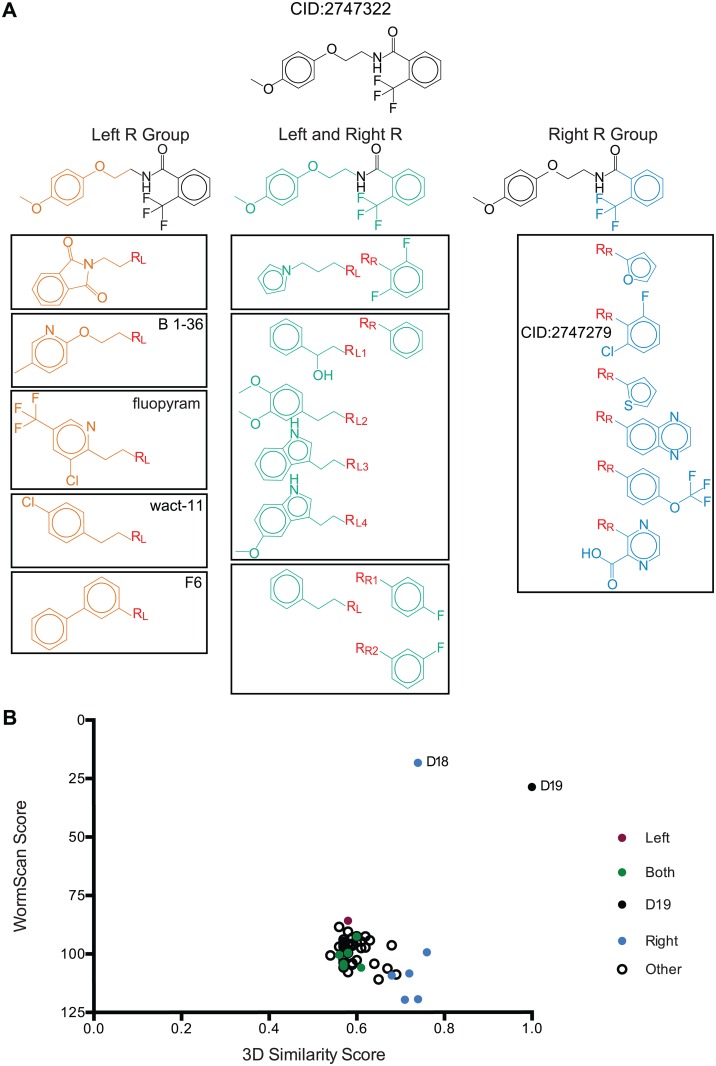
A structure-activity relationship (SAR) analysis of CID 2747322. (A) *Hierarchical maps*. Hierarchical maps were used to categorize 14 of the 50 compounds from the SAR screen, with variations in the right R-group 2-(trifluoromethyl)benzene, left R-group 2-(4-methoxyphenoxy)ethyl or both R-groups. Included in the hierarchical map is a Bayer patented compound (N-(2-((5-methylpyridin-2-yl)oxy)ethyl)-2-(trifluoromethyl)benzamide) [65], which has a 3D similarity score of 0.82. Fluopyram has a 3D similarity score of 0.58. Also included is the WACT-11 compound identified by Burns [34] with a 3D similarity score of 0.66, and the flutolanil analogue (CID 49852661) used for modeling which has a 3D similarity score of 0.60. (B) *A SAR analysis for CID 2747322 against our internal library of 24*,*989 compounds from Maybridge and Chembridge libraries*. The top 50 compounds with the highest 3D similarity scores to CID 2747322, calculated using Screen3D version 2015 [57] were re-pinned for two additional biological replicates. Compounds with variations on the left R-group 2-(trifluoromethyl)benzene or the right R-group 2-(4-methoxyphenoxy)ethyl or both R-groups are highlighted. The compound CID 2747279 (3D Similarity Score of 0.74), has variations on the right R-group was the only compound from the set of 50 that displayed strong anthelmintic activity.

A SAR approach was undertaken for CID 2747322 by considering the 26,000 screened compounds to gain insight into the structural moieties of CID 2747322 important for biological activity and to identify potential avenues of new medicinal chemistry [78]. This SAR exploration can provide insight into what changes in the functional groups of CID 2747322 can be tolerated while still maintaining anthelmintic activity and can guide future medicinal chemistry studies to enhance the activity. The compounds from the SAR analysis are highlighted with respect to changes in their R-group(s) compared to CID 2747322 (Fig 4a). Compounds with changes on the right R-group are grouped based on their 3D similarity score and depicted in blue. Only one compound from the set had a change restricted to the left R-group, whereas the majority of the CID 2747322 analogs display changes in both the left and right R-groups. Changes on the right and left R-groups of CID 2747322, 2-(trifluoromethyl)benzene and 2-(4-methoxyphenoxy)ethyl respectively, were used to group the most similar compounds of CID 2747322 into a hierarchical map (Fig 4a). The patented Bayer AG molecule 1–36 was found to cluster closely to CID 2747322 with a 3D similarity score of 0.82. Additionally, the anthelmintic WACT-11 compound [34] also clusters to CID 2747322 with a 3D similarity score of 0.66. The flutolanil analog N-biphenyl-3-yl-2-(trifluoromethyl)benzamide, an established complex II inhibitor, also has a 3D similarity to CID 2747322 of 0.60. The Bayer compound fluopyram [66], recently approved as an anthelmintic has a 3D similarity score of 0.58. These molecules have a common peptide–CO–NH–linker.

The 50 compounds from our survey with the greatest 3D similarity scores to CID 2747322 were retested for biological activity against *C*. *elegans*. Often, structurally similar molecules are found to have similar activity [79]. As in the original screen only one of these 50 related compounds has strong anthelmintic activity in *C*. *elegans*. This compound is the primary hit CID 2747279 (3D Similarity Score of 0.74; Fig 4b). Our analysis using SAR and drug retesting indicates that only variations in the right R-group of CID 2747322 maintain anthelmintic activity and that no substituents at the left R-group are tolerated. Based on the predicted chemical properties of these substituents we can speculate as to why certain structures are active while others are not. For example, the electron-withdrawing property of CID 2747322 right R-group 2-(trifluoromethyl) benzene induces an uneven distribution of electric charge. In this scenario, the R-group takes on a partial negative charge and the benzene ring becomes an electron-deficient π molecular orbital. This is also found in the right R-group of CID 2747279 and not with the furan right R-group, which was actually scored to be more similar with Screen3D. Other R-group variations did not have any anthelmintic activity, including the substitution of a large hydrophobic quinoxaline group. This anthelmintic specificity of CID 2747279 and CID 2747322 suggests that the binding pocket of *C*. *elegans* mitochondrial complex II is responsible for the biological activity.

### Modeling CID 2747322 binding to mitochondrial complex II quinone pocket

**Fig 5:**
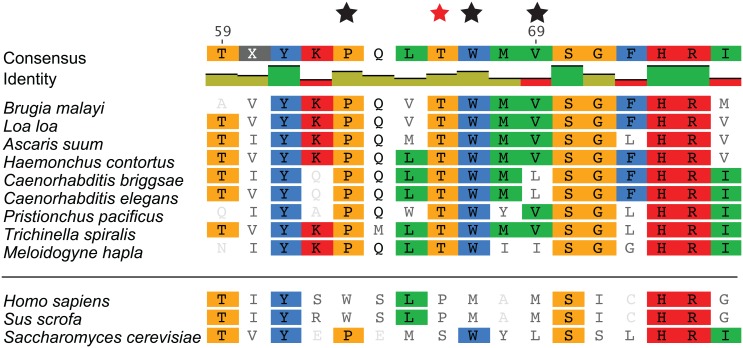
A phylogenetic comparison of the quinone binding site in MEV-1. Sequences for *mev-1* of representative nematode species together with, *Homo sapiens*, *Sus scrofa*, and *S*. *cerevisiae* were obtained using BlastP and aligned by ClustalW. The key residues involved in quinone binding, are conserved amongst all nematode species examined. The red star indicates the T66I variant that results in resistance to CID 2747322 exposure in the VC3631 strain. Residues important for the left binding pocket of CID 2747322 are indicated with a black star. Image was generated using Geneious version 8.1.7.

**Fig 6:**
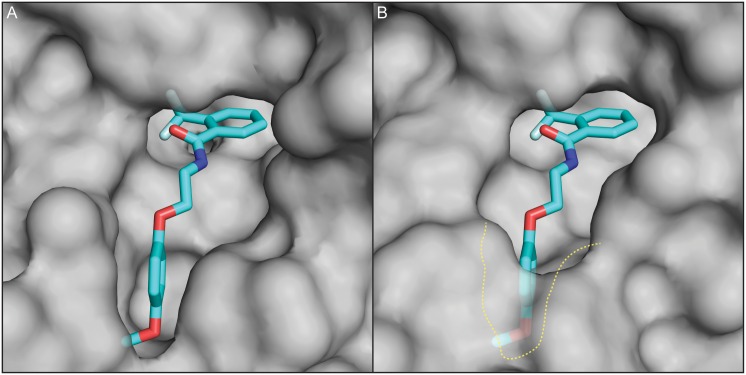
Complex II specificity model of CID 2747322 surface representation of nematode and vertebrate homologue. (A) The *A*. *suum* structure of succinate dehydrogenase in complex II of the mitochondria, illustrating how the CID 2747322 molecule can be accommodated in the quinone binding pocket. The molecule CID 2747322 was aligned to analogue flutolanil using Screen3D [76]. The right side of CID 2747322, 2-(trifluoromethyl)benzene is found binding in a hydrophobic pocket with a Cation–π interaction from Arg278. The left side of CID 2747322, 2-(4-methoxyphenoxy)ethyl is found to orientate within a hydrophobic pocket. (B) The porcine structure of succinate dehydrogenase in complex II of the mitochondria indicates a non-favorable binding of the CID 2747322 molecule. The left side of CID 2747322 is unable to orient within the smaller hydrophobic pocket. Highlighted in yellow dots is the larger pocket of the nematode binding pocket.

We leveraged the fact that the antifungal compound flutolanil is an excellent inhibitor of *A*. *suum* mitochondrial complex II, which has previously been shown to bind within the quinone pocket of the crystal structure of *A*. *suum* complex II (PDB: 4YTM) [76] to further explore the compound-target interaction. CID 2747322 has structural similarity to the flutolanil analogue N-biphenyl-3-yl-2-(trifluoromethyl)benzamide (Fig 4a). Furthermore, the *A*. *suum* mitochondrial complex II has conserved sequence homology with *C*. *elegans* (Fig 5), making it an excellent template for 3D homology modeling for *C*. *elegans*. To better understand how CID 2747322 interacts with the target site, we constructed a model of the *C*. *elegans* succinate dehydrogenase complex in which the flutolanil analogue is used to model the CID 2747322 3D position within the *C*. *elegans* protein model (Fig 6A). The docked position of CID 2747322 was found to form essential non-covalent interactions annotated in the structure of flutolanil and *A*. *suum* complex II (PDB: 4YTM) [76]. The *C*. *elegans* homology structure shared the conserved TRP197 of SDHD-1, which forms a hydrogen bond with the amide group of CID 2747322. Additionally, the ARG89 of SDHB-1 can form a cation–π interaction with the right R group 2-(trifluoromethyl)benzene of CID 2747322. The left R group of CID 2747322 (2-(4-methoxyphenoxy)ethyl) docks within the hydrophobic pocket between complex II subunits SDHB-1 and SDHC-1.

While flutolanil is a potent *A*. *suum* complex II inhibitor (IC_50_ 0.0245 μM), it has been shown to be a less effective inhibitor of the porcine complex II (IC_50_ 8.61 μM) [80]. The aligned vertebrate porcine protein structure of complex II lacks a hydrophobic pocket for the left R group of D19 (Fig 6B). The key residues important for this binding pocket are the proline 63, tryptophan 68 and leucine 69 of MEV-1 in *C*. *elegans*. These critical residues of MEV-1 are present in free-living as well as plant and animal parasitic nematodes (Fig 5). These sites are not conserved in vertebrates, and a hydrophobic pocket for the left R group is therefore not available for docking. Additionally, CID 2747322 binding does not form interactions with the mammalian TRP197 of SDHD-1 and Arg89 SDHB-1 as favorable as those it forms with analogous residues in *C*. *elegans*, consistent with our observation that CID 2747322 is a more potent inhibitor of *C*. *elegans* than of mammalian cells in culture (Table 1). These structural differences may identify the key drug-target interactions responsible for the specificity of CID 2747322 in nematodes, and its lack of mammalian cell activity.

## Discussion

The need for new anthelmintics has been voiced by WHO and echoed by many other organizations, notably the Gates Foundation. However, this is no small task [81]. To identify a nematicide that is not also a biocide is difficult enough, but then to discover a compound that does not engage biochemical pathways targeted by currently used anthelmintics is another order of difficulty. Success will depend on screening a sufficiently large swath of chemical space quickly and efficiently and it requires a test organism that provides the means to identify pathways and discriminate among modes of biochemical action. The screening platform describe here meets these various criteria. The flatbed scanner method is quick and efficient [46]. As WormScan is also inexpensive it is relatively easy to scale up screening capacity through parallel processing to be truly high throughput, for example, by permitting screens of pharmaceutical chemical libraries of hundreds of thousands of synthetic or natural compounds. In addition, the reporter organism, *C*. *elegans*, is a good surrogate for parasitic nematodes [82]. The genome content has considerable overlap with many parasitic nematodes as does its physiology, but unlike most parasitic nematodes it is easy to grow in large numbers and there is an extensive genetic and molecular toolkit to tease apart the mode of action of any new potential anthelmintic (reviewed in [23]). Furthermore, this platform is flexible. Incorporating reporter proteins or other readouts of phenotypic effects can be accommodated with the existing detection system, and refinements such as higher resolution imagers could further expand the repertoire of phenotypes that are assayable. We would stress that what we are addressing here is the issue of replenishing the pool of compounds that may be potential anthelmintics. As those working in the area of animal health discovery well know this is only the first step in a long process of drug discovery and drug development before commercial use and deployment.

Our screen and data analysis allowed us to identify 14 new compounds that affect *C*. *elegans* growth and fecundity. Two of these compounds are in the same chemical class as flutolanil and fluopyram (Fig 4a). Not all of these compounds are nematode specific, but our analysis suggests that nematodes are more sensitive to the action of at least five of the compounds than are other organisms (Table 1). We also demonstrated that three of these five compounds do not act through the biochemical pathways targeted by the known anthelmintics ivermectin, benomyl. levamisole or amino acetotnitrile (the other two compounds have not yet been tested). Thus parasites present in the wild resistant to these anthelmintics should not be resistant to this new chemistry. Two of these compounds do appear to act through a pathway common to other complex II anthelmintics including fluopyram. While these compounds satisfy one of the major criteria necessary for any new anthelmintic: phylogenetic specificity, they do not all have a novel biochemical mode of action. Nevertheless, these compounds may eventually have applications for agricultural, livestock or even human nematode parasites.

For the compound with the greatest differential effect in our phylogenetic assay, CID 2747322, we identified two separate resistant nematode lines after mutagenesis and screening. One line contains an alteration in *pink-1*, the nematode homolog of PINK1, a serine/threonine kinase involved in monitoring mitochondrial homeostasis. The second line contains an alteration in the gene *mev-1*, the nematode ortholog of a succinate dehydrogenase cytochrome b560 subunit. This protein is an integral membrane protein and is part of the mitochondrial respiratory chain complex II. The MEV-1 protein is required for oxidative phosphorylation.

It is probably no coincidence that both genes are involved in the function of mitochondria, and this may be the key to understanding resistance to CID 2747322. In both examples, resistance to the compound appears to require quite specific mutations. For example, loss of PINK-1 function does not offer resistance to the compound, only a subset of missense mutations confer resistance, and many of the MEV-1 amino acid alteration cluster around a quinone binding site. PINK-1 resistance may be a form of indirect resistance but at present we cannot explain how this may occur. From our analysis of mtDNA copy number in resistant strains the mechanism of resistance does not appear to act strictly through altering copy number.

We are on somewhat better footing in regard to interpreting the relationship between *mev-1* function and its resistance, when mutated, to CID 2747322. Combining our structural analysis of the succinate dehydrogenase complex with those of Burns *et al* [35] on resistance to WACT-11 suggests that this complex is the direct target of both CID 2747322 and WACT-11. Our *gk3613* mutation is identical to one of the many mutations isolated for WACT-11 resistance, and this mutation affects the quinone binding pocket of the complex. The Burns *et al* group identified mutations in all the subunits of succinate dehydrogenase and most cluster around these amino acids. Guided by their observations, and since we only identified a single mutation in *mev-1*, we took advantage of the Million Mutation Project to test a number of mutant alleles for all four units of succinate dehydrogenase. From this unbiased testing (i.e. no prior selection for resistance) we identified mutations throughout the protein that confer resistance to CID 2747322.

Our 3D modeling of the binding of CID 2747322 to the quinone pocket suggests that resistance occurs by altering the pocket just enough to prevent the binding of the anthelmintic but not of quinone. Using this information we could also model why the succinate dehydrogenase complex from mammals is not sensitive CID2747322. The quinone pocket of the mammalian complex is altered just enough to prevent CID2747322 binding, thus conferring natural resistance. Importantly, these findings demonstrate that our post-screening analytic approach using the power of *C*. *elegans* genetics coupled with the MMP data makes it possible to rapidly generate important mechanistic information for previously uncharacterized compounds.

The mitochondrial complex II appears to be a particularly engageable target for small molecule binding. Structurally similar inhibitors have been characterized by three independent groups as anthelmintic agents (Bayer AG [65], the Inaoka group [80] and the laboratory of Peter Roy [34]). Indeed the newly certified Bayer manufactured anthelmintic fluopyram is based on this same chemical backbone and mode of action. We independently discovered the same class of complex II inhibitors and further showed that CID 2747322 can be docked into the worm complex II structure using the molecular modeling approach reported by Inaoka DK *et al* [80]

In this study we reaffirm what other recent studies have demonstrated; *C*. *elegans* is an excellent model for anthelmintic discovery and characterization. Granted there is much to do after a potential anthelmintic is identified but these results are encouraging first steps and suggest that the *C*. *elegans* model provides a potential solution to replenishing the early stage pipeline for anthelmintics. One clear lesson from our study is that it can be difficult to exclude redundancy of effort. Between our group and the Burns *et al* study we have examined almost 93,000 compounds. Also, between the compound libraries screened in the two studies there is only an overlap of 1,769 compounds. However, we still converged onto a common chemical backbone with one of our most promising hits. As these libraries are commercially available there is nothing to prevent others from discovering further redundancy. More encouragingly, a comparison of the hits from our screen (14 molecules) to those identified in the Burns *et al* study reveals that while nine of our hits fall into clusters identified in the previous study, five are novel chemical identities (see S2 Table). We should point out that even those hits falling into the same cluster are not the identical molecule.

In future we will be exploring more natural compound libraries. After all, this is the origin of the avermectin family of compounds, first identified in the bacterium *Streptomyces avermitilis*, and these have proven to be the most effective anthelmintics for the past forty years (reviewed in [83], [84]; also see [85]). In future the usefulness of our approach will be evaluated by efficacy studies, including tests of compound hits in parasitic nematode models [86–88].

## Supporting Information

S1 Fig Genetic mapping and Whole Genome Sequencing procedures used to identify region conferring drug restiance. The *C*. *elegans* strain DM7448 was used to map and identify ethyl methanesulfonate (EMS) induced mutation linked to the drug resistance. DM7448 provides a visual marker in the form of muscle wall GFP to ensure the progeny have been successfully outcrossed. Four outcrosses would commonly result in replacement of unrelated chromosome and would be expected to also remove many of the unrelated mutations by recombination around the causal mutation. After each outcross, worms were re-tested for drug resistance to ensure homozygosity of the mutation responsible for drug resistance. The mutation responsible for resistance will be associated within a region of unrelated mutations that are unlikely to be removed after out-crossing because of their proximity to the drug resistance mutation. DNA is extracted from one recombinant after the last round of outcrossing and sequenced to ≥ 15x coverage to identify the candidate mutations responsible for drug resistance.
(EPS)

S2 Fig Developmental stage of inhibition in *C*. *elegans* after CID 2747322 exposure. Ten VC2010 L1 stage *C*. *elegans* were sorted into a DMSO control well or 90 μM of CID 2747322. Nematodes were removed from the DMSO control or CID 2747322 after 24/48/72 hours and allowed to recover for 30 minutes on standard agar plates.
(TIFF)

S3 Fig Development of *C*. *elegans* after removel from CID 2747322. Greater than 50 VC2010 L1 stage *C*. *elegans* were sorted into a DMSO control well or 90 μM of CID 2747322. Nematodes were removed from DMSO control or CID 2747322 after 2 days of exposure and left to recovery for 24/48/72 hours.
(TIFF)

S4 Fig Dosage curve for effects on *C*. *briggsae* of CID 2747322. Two L4 *C*. *briggsae* animals were placed into each well of a 96-well pate for five days of exposure to CID 2747322. The IC_50_ value of 16 μM was calculated using Mathematica 8.0.
(PDF)

S5 Fig Dosage curve for effects on *Meloidogyne hapla* of CID 2747322. The percentage of immobilization of infective juveniles of *Meloidogyne hapla* was measure after ten days of exposure to the compound. Data are from two separate trials testing 0–320 and 0–160 μM concentration ranges, respectively, with each point representing the mean of twelve test wells.
(PDF)

S1 Table Manual screen of a set of 3,584 known compounds. (XLSX)

S2 Table Compound assignment to structural groups. (DOCX)

S1 Software Standalone Java Program. (ZIP)
