# Supplementary figures and images for: Using C. elegans Forward and Reverse Genetics to Identify New Compounds with Anthelmintic Activity
Source: PLoS Negl Trop Dis. 2016 Oct 18;10(10):e0005058. doi: 10.1371/journal.pntd.0005058 (PMC5068747; doi:10.1371/journal.pntd.0005058)

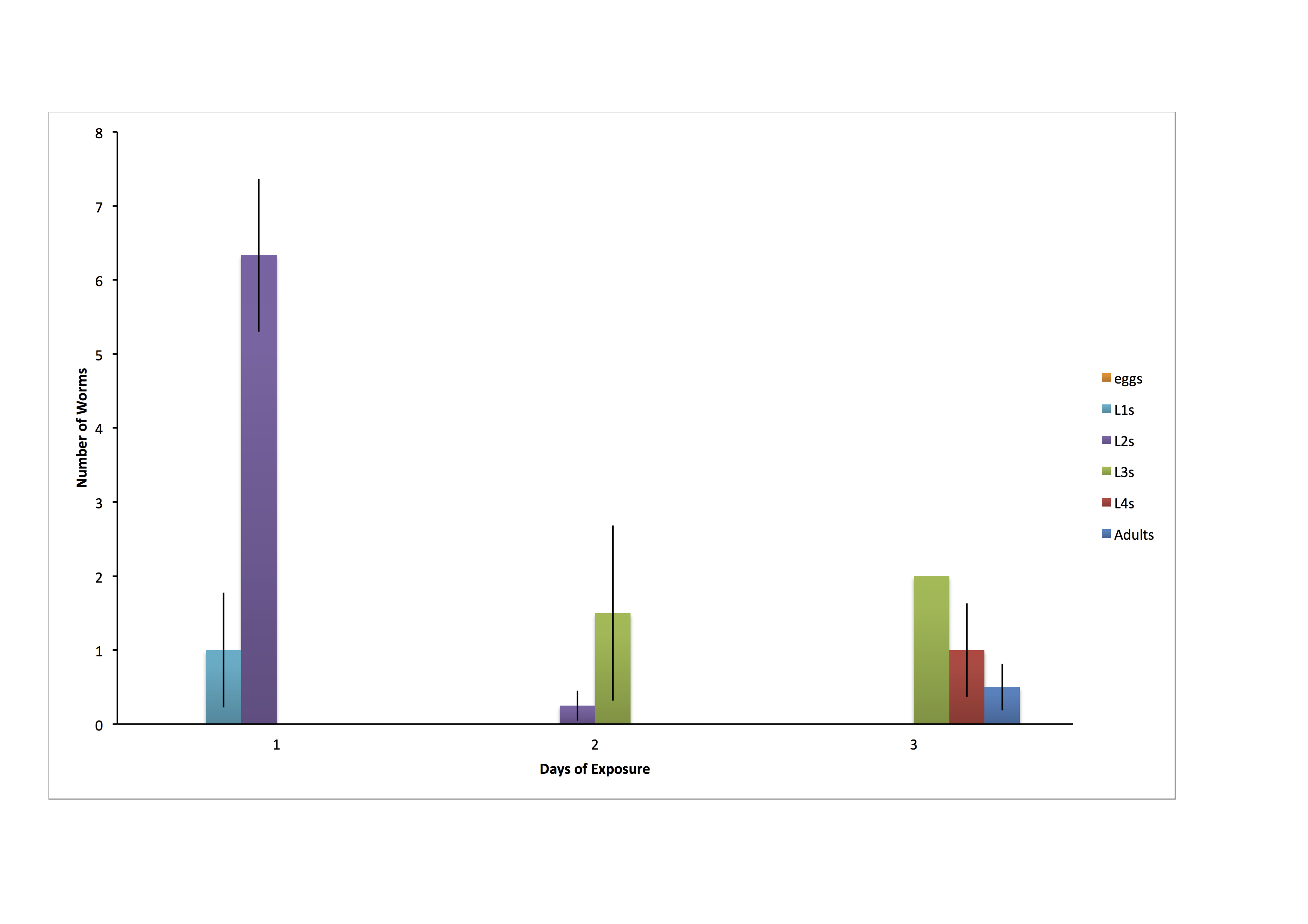

Supplement: S2 Fig — Ten VC2010 L1 stage C. elegans were sorted into a DMSO control well or 90 μM of CID 2747322. Nematodes were removed from the DMSO control or CID 2747322 after 24/48/72 hours and allowed to recover for 30 minutes on standard agar plates. (TIFF) [file pntd.0005058.s002.tiff]

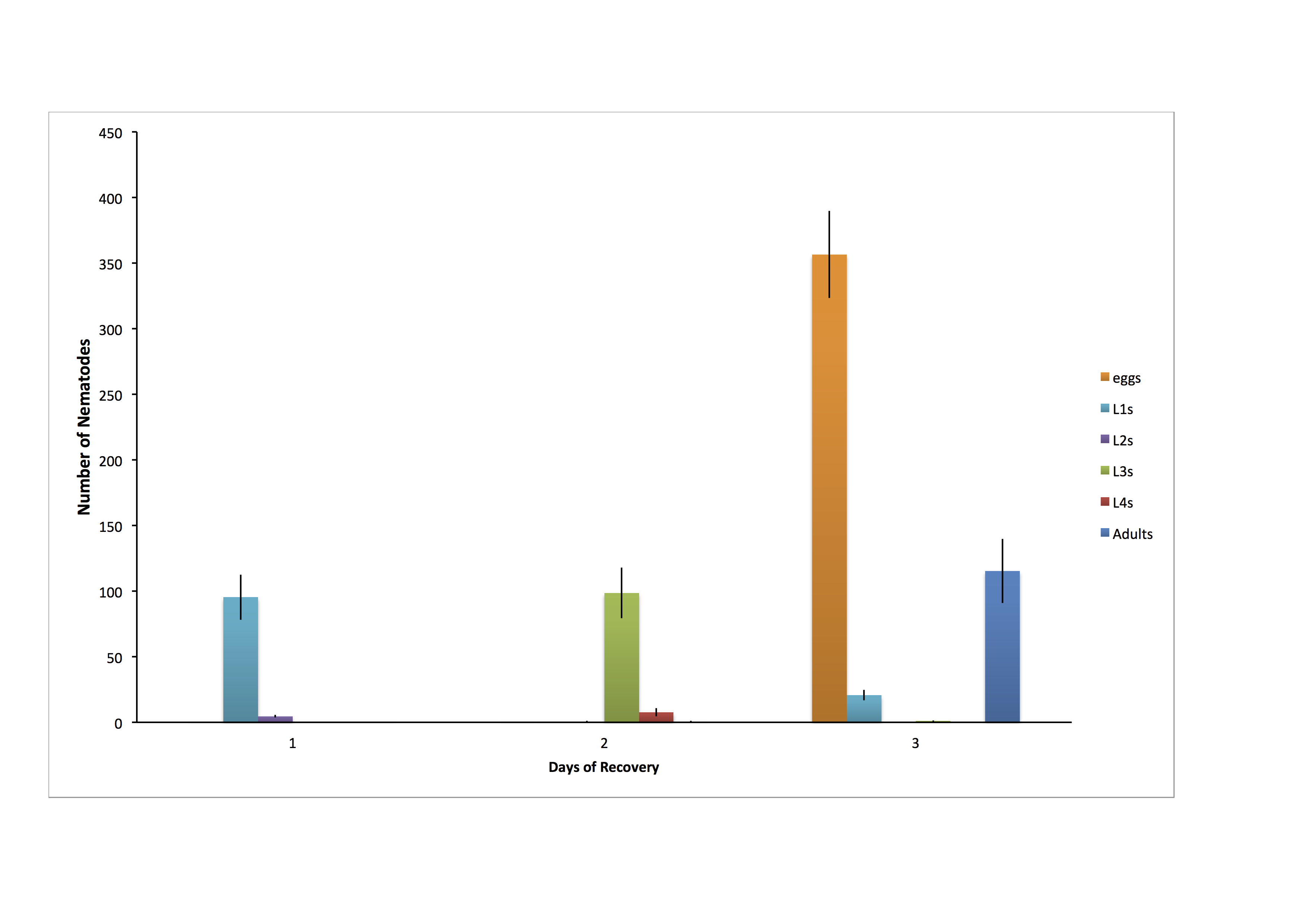

Supplement: S3 Fig — Greater than 50 VC2010 L1 stage C. elegans were sorted into a DMSO control well or 90 μM of CID 2747322. Nematodes were removed from DMSO control or CID 2747322 after 2 days of exposure and left to recovery for 24/48/72 hours. (TIFF) [file pntd.0005058.s003.tiff]

CID 2747322 (Log10  $\mu$ M)

WormScan Score

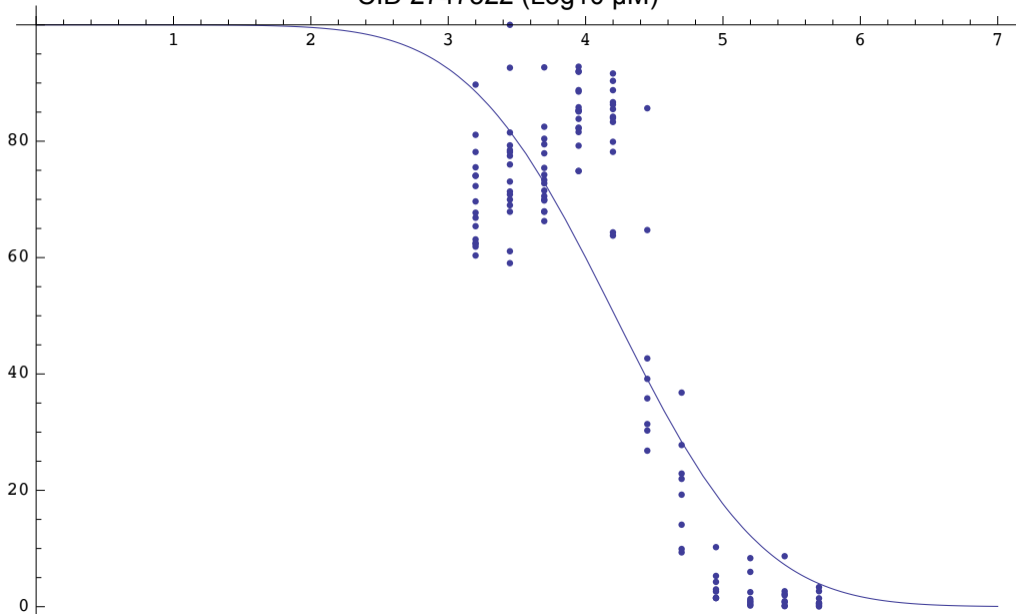

Supplement: S4 Fig — Two L4 C. briggsae animals were placed into each well of a 96-well pate for five days of exposure to CID 2747322. The IC50 value of 16 μM was calculated using Mathematica 8.0. (PDF) [file pntd.0005058.s004.pdf]

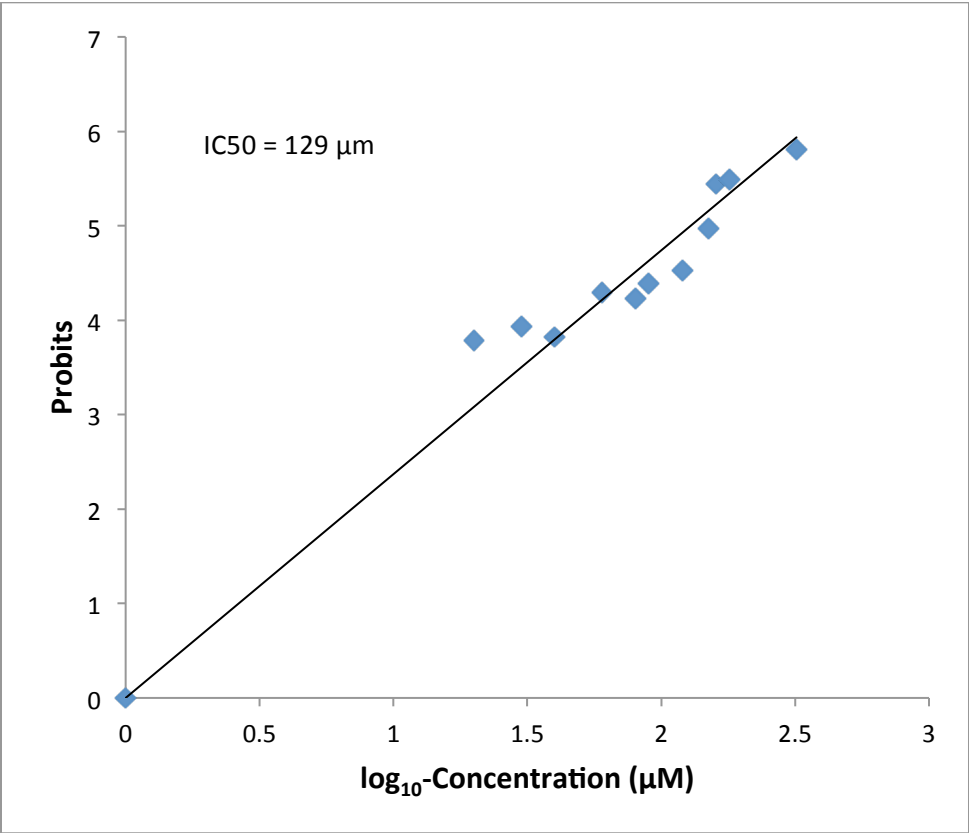

Supplement: S5 Fig — The percentage of immobilization of infective juveniles of Meloidogyne hapla was measure after ten days of exposure to the compound. Data are from two separate trials testing 0–320 and 0–160 μM concentration ranges, respectively, with each point representing the mean of twelve test wells. (PDF) [file pntd.0005058.s005.pdf]
