# Supplementary material for: Using C. elegans Forward and Reverse Genetics to Identify New Compounds with Anthelmintic Activity
Source: PLoS Negl Trop Dis. 2016 Oct 18;10(10):e0005058. doi: 10.1371/journal.pntd.0005058 (PMC5068747; doi:10.1371/journal.pntd.0005058)
Supplement: S2 Table — (DOCX) [file pntd.0005058.s007.docx]

| Drug | 2D Structure | Burns structural group | 2D Most similar WACT compound Tanimoto  score |
| --- | --- | --- | --- |
| D1 CID 3254982 | 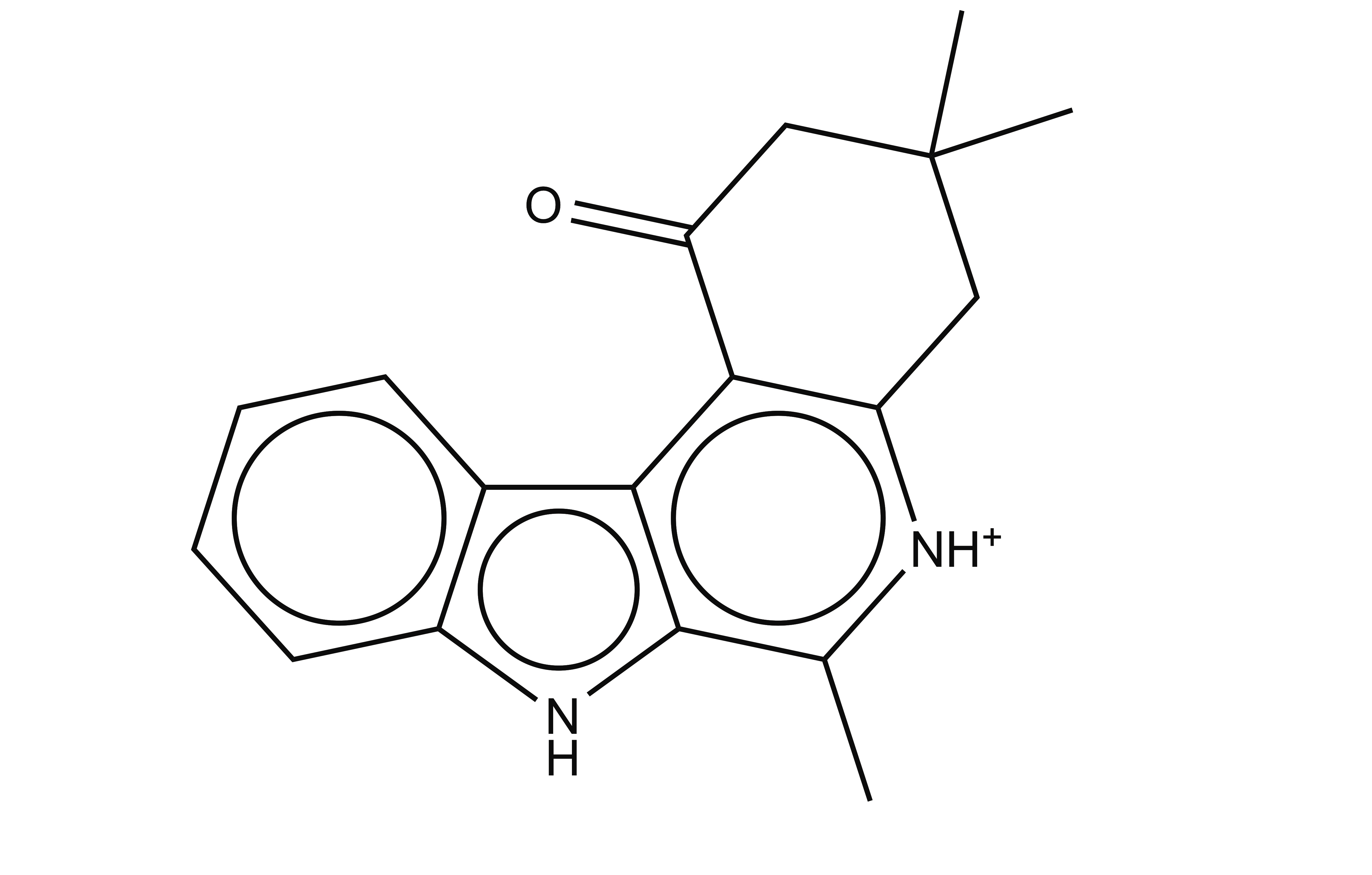 | Cluster 11 | 0.64  WACT42 |
| D2 CID 2238042 | 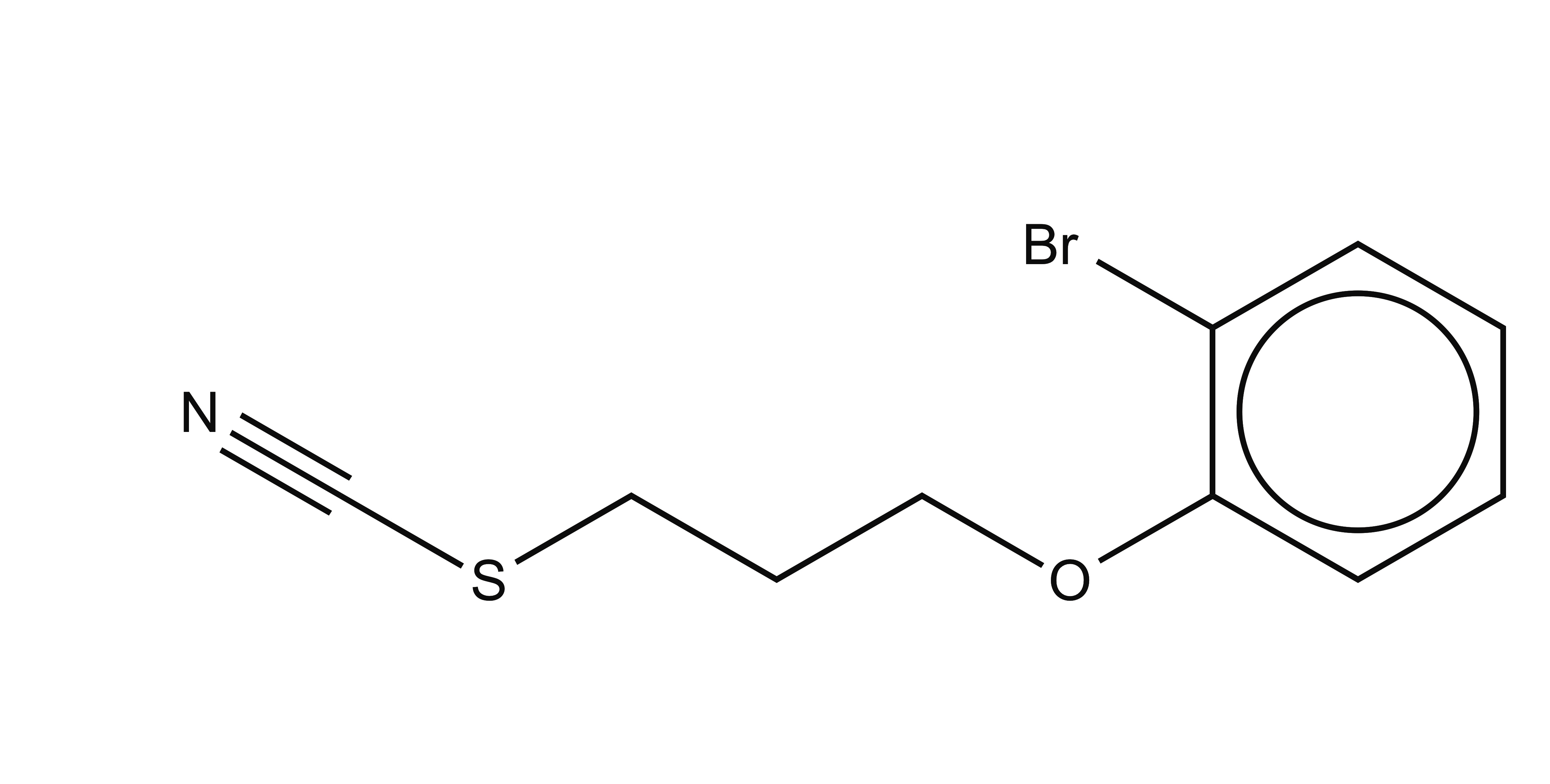 | N/A | N/A |
| D4 CID 796072 | 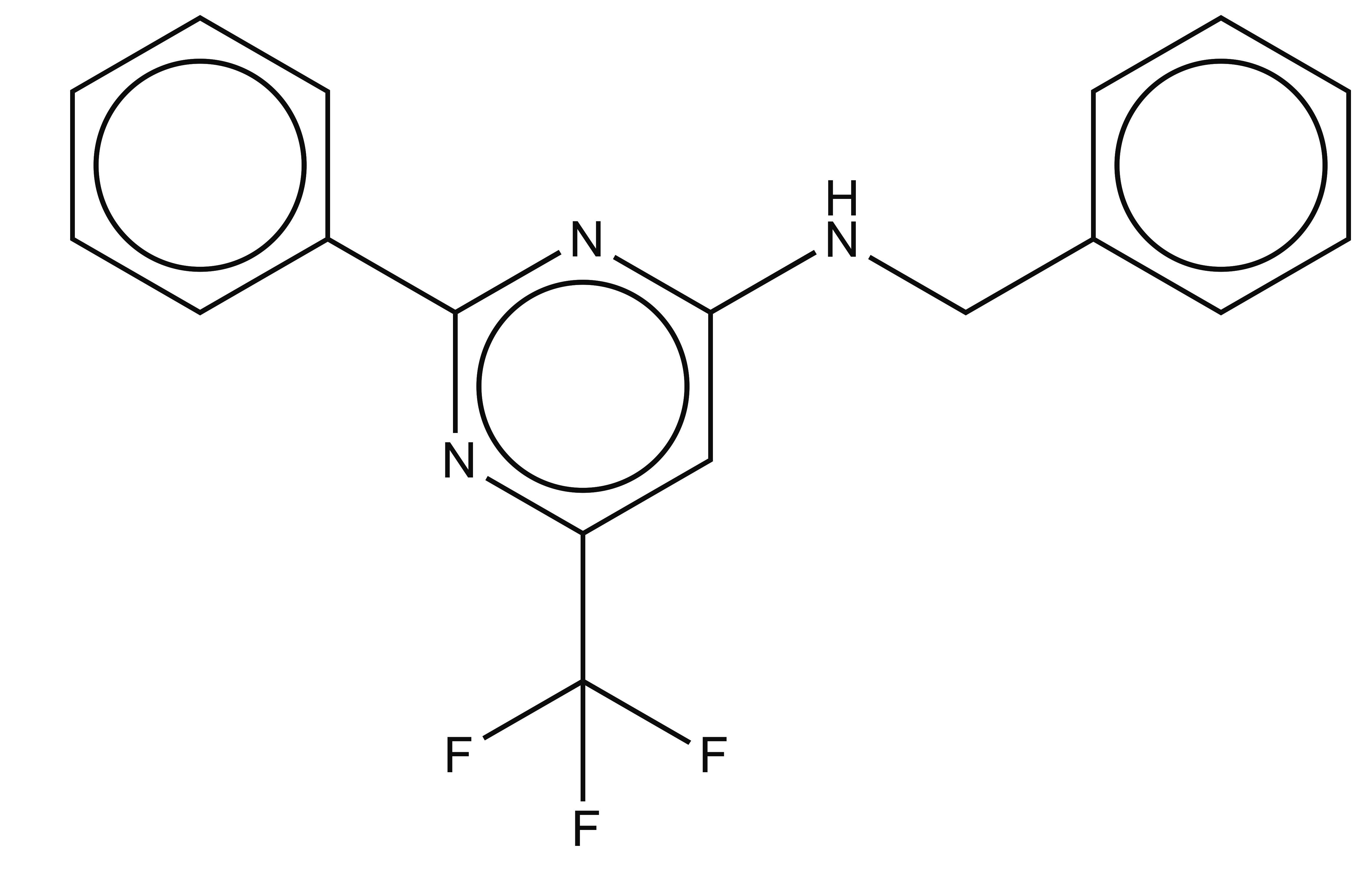 | No Cluster | 0.60  WACT189 |
| D5 CID 6456299 | 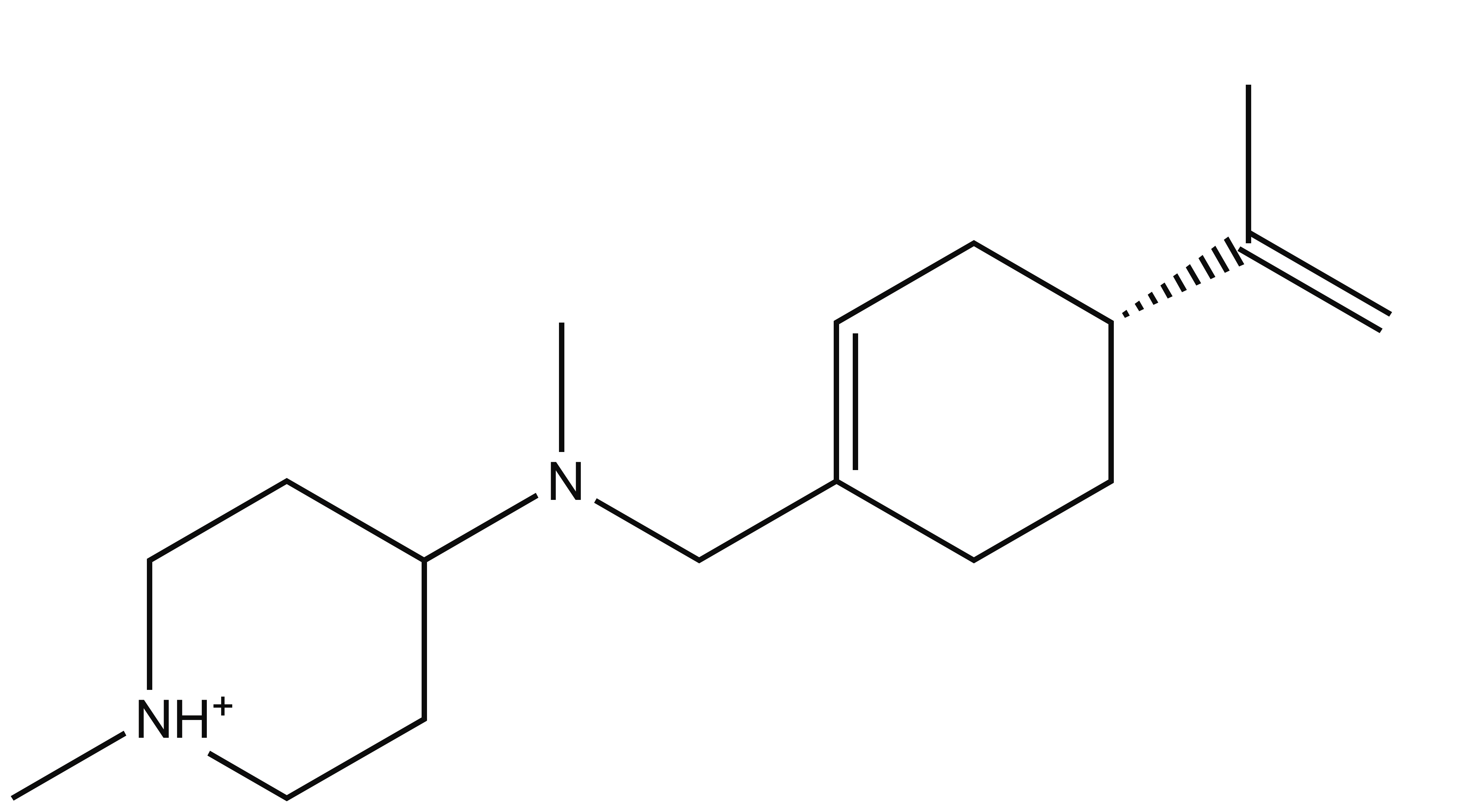 | N/A | N/A |
| D6 CID 766260 | 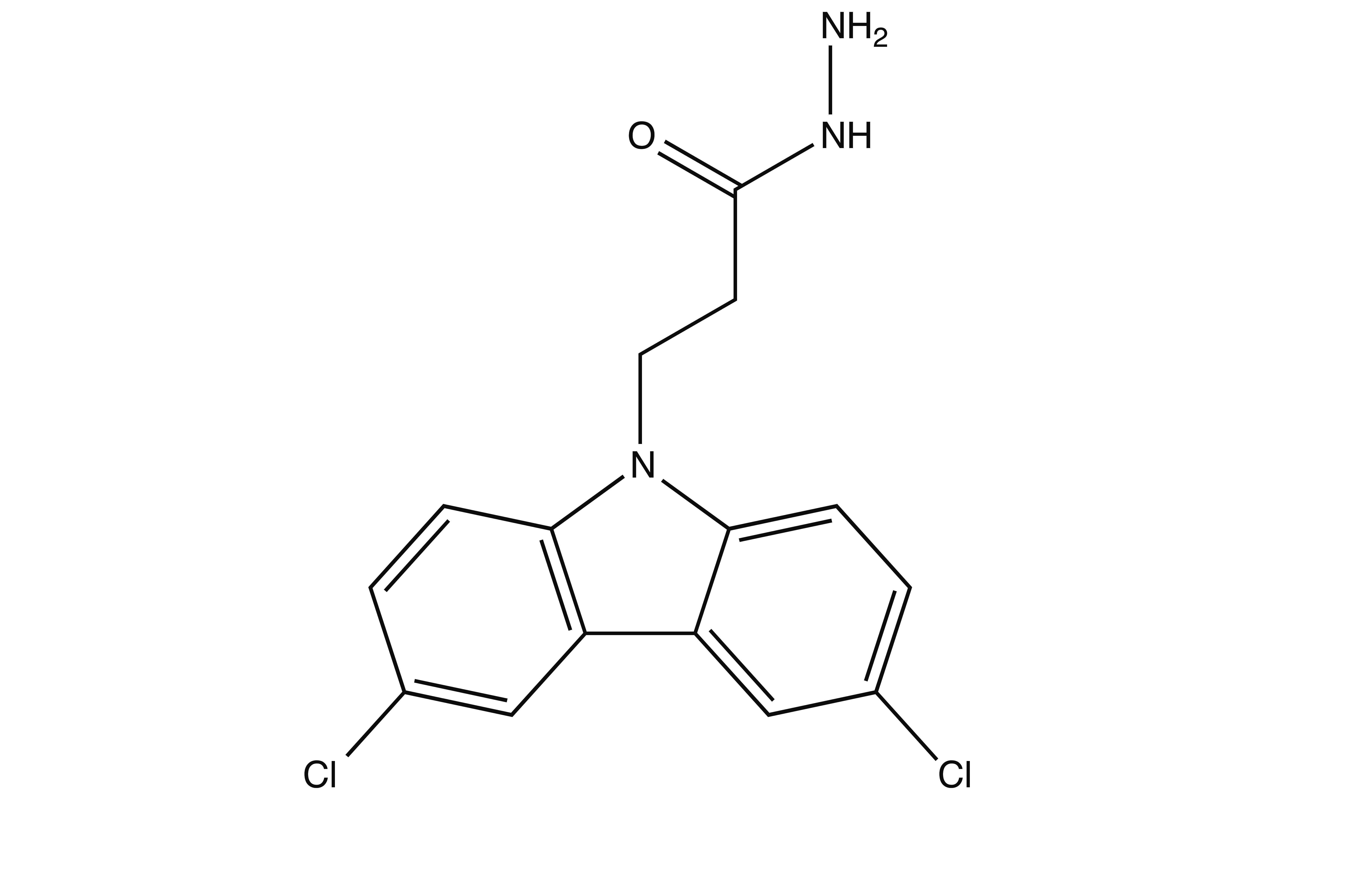 | No Cluster | 0.63  WACT35 |
| D7 CID 2222671 | 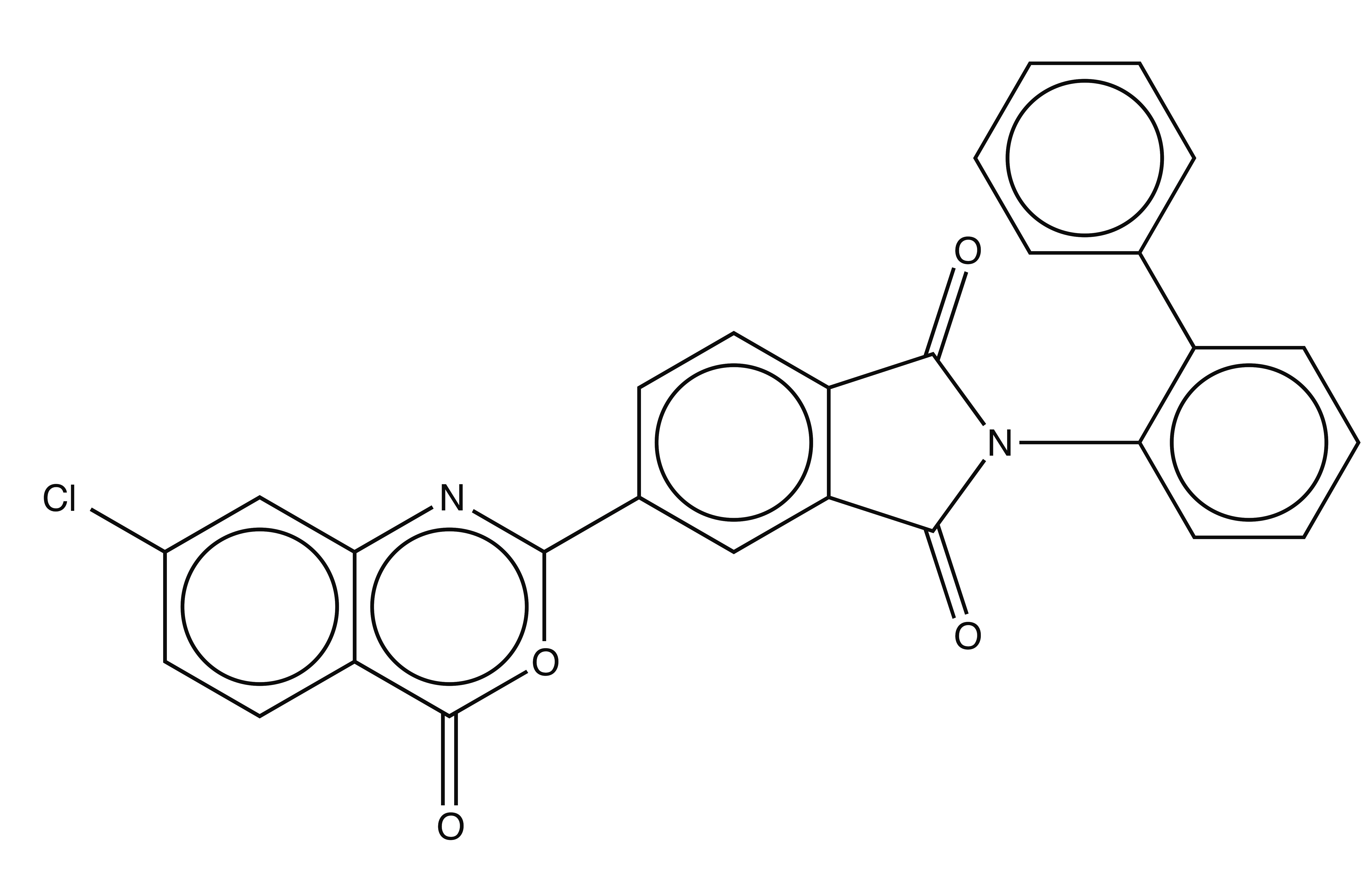 | No Cluster | 0.61  WACT137 |
| D8 CID 6741218 | 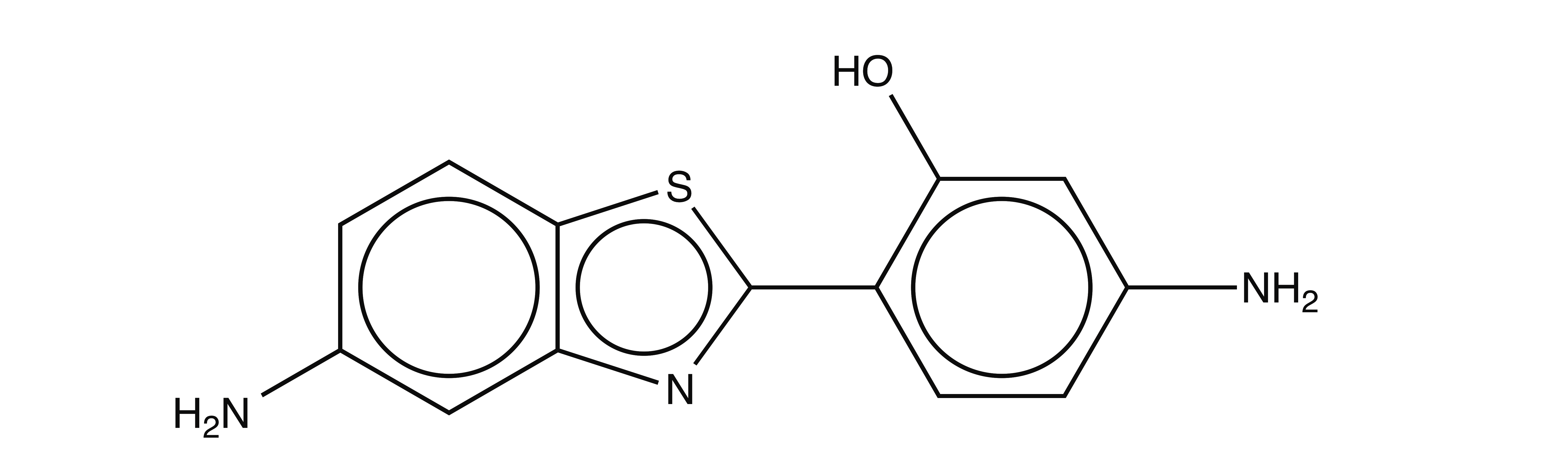 | Cluster 3 | 0.93  WACT162 |
| D10 CID 6745334 | 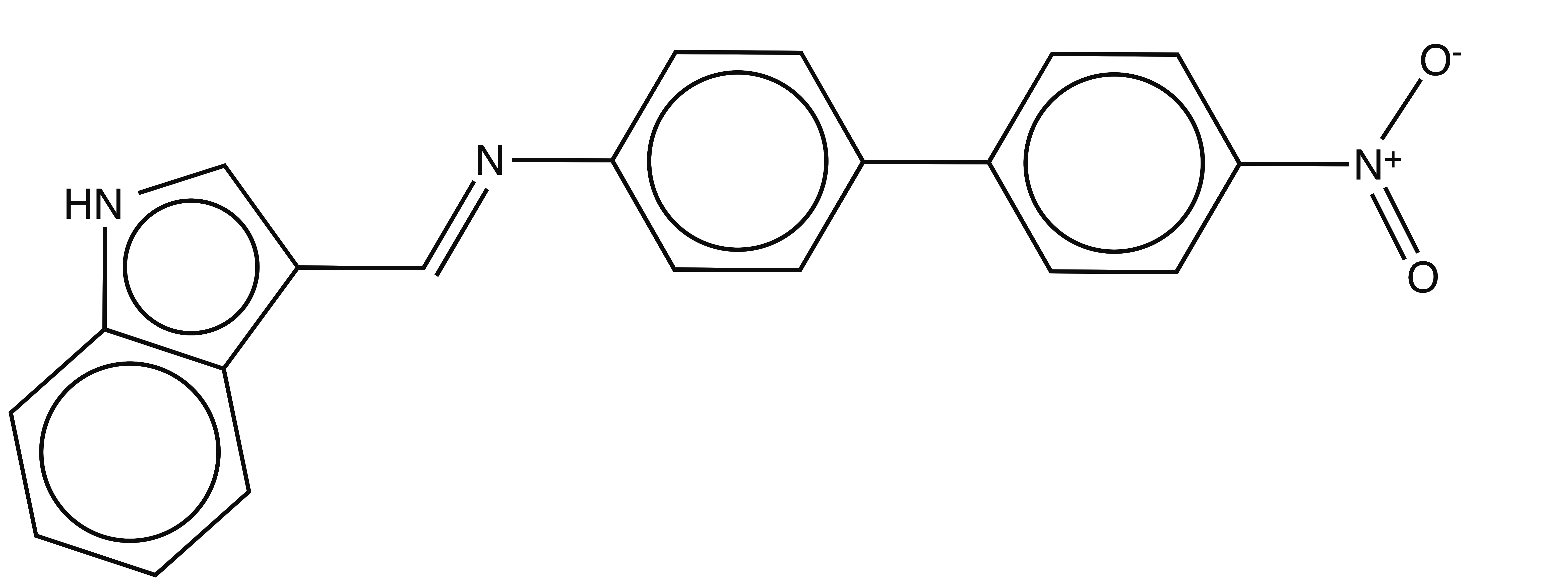 | No Cluster | 0.56  WACT394 |
| D13 CID 2743002 | 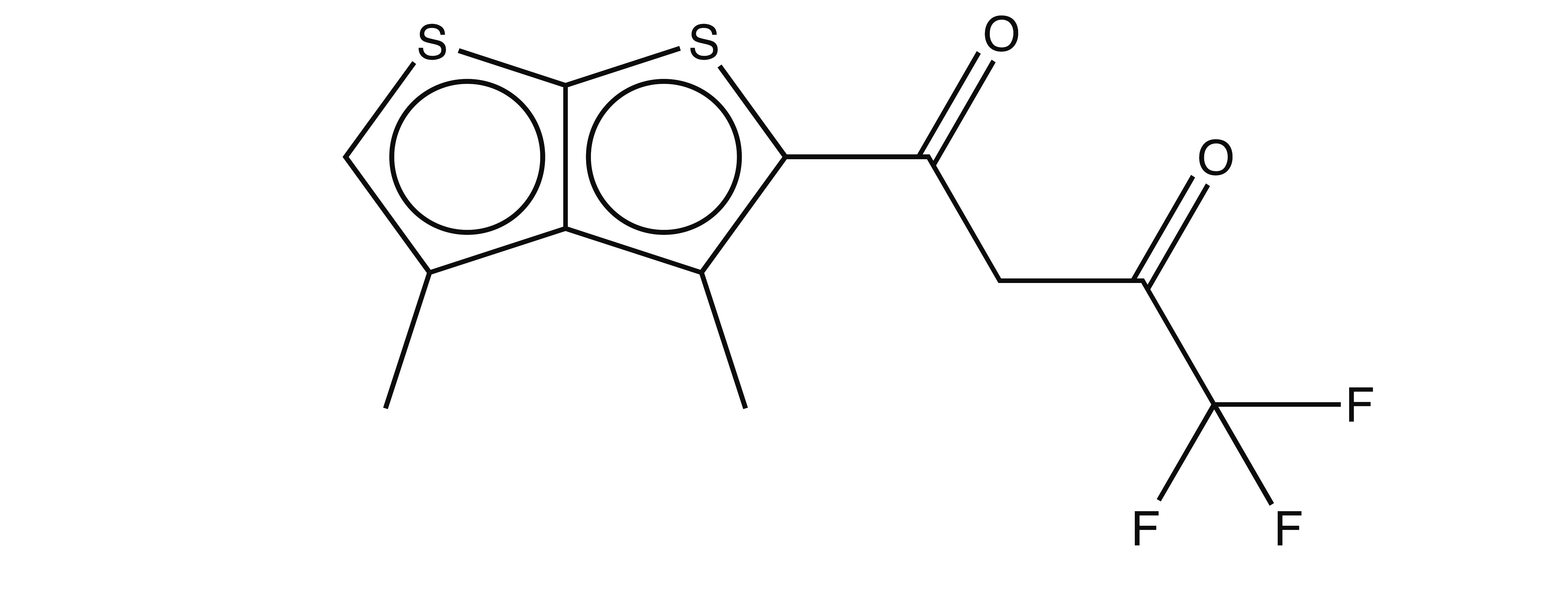 | N/A | N/A |
| D15 CID 2823320 | 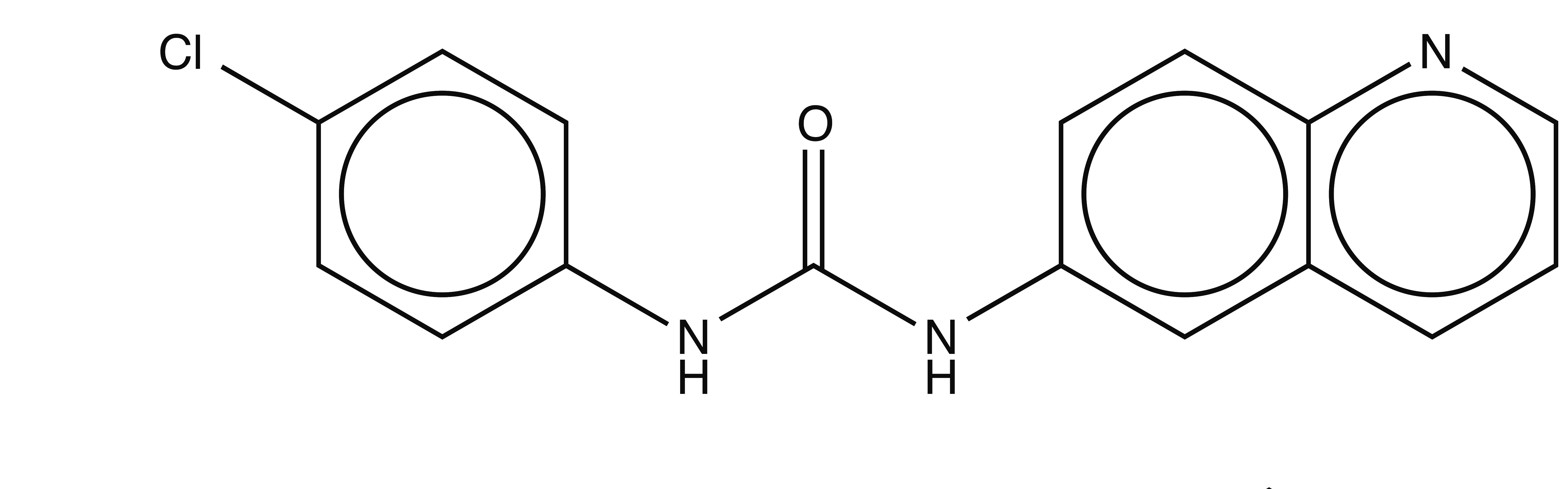 | N/A | N/A |
| D16 CID 2740991 | 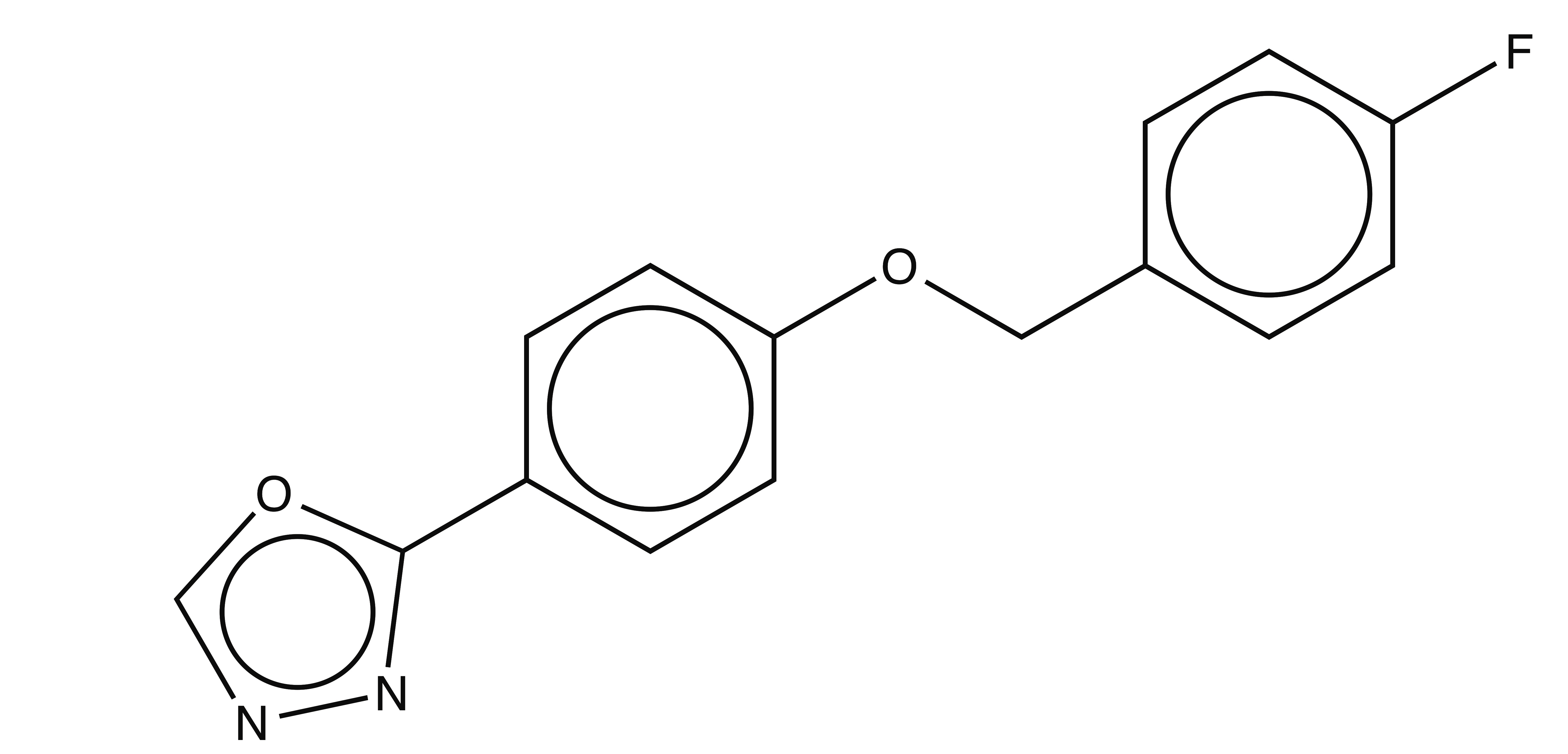 | No Cluster | 0.61  WACT210 |
| D17 CID 2745791 | 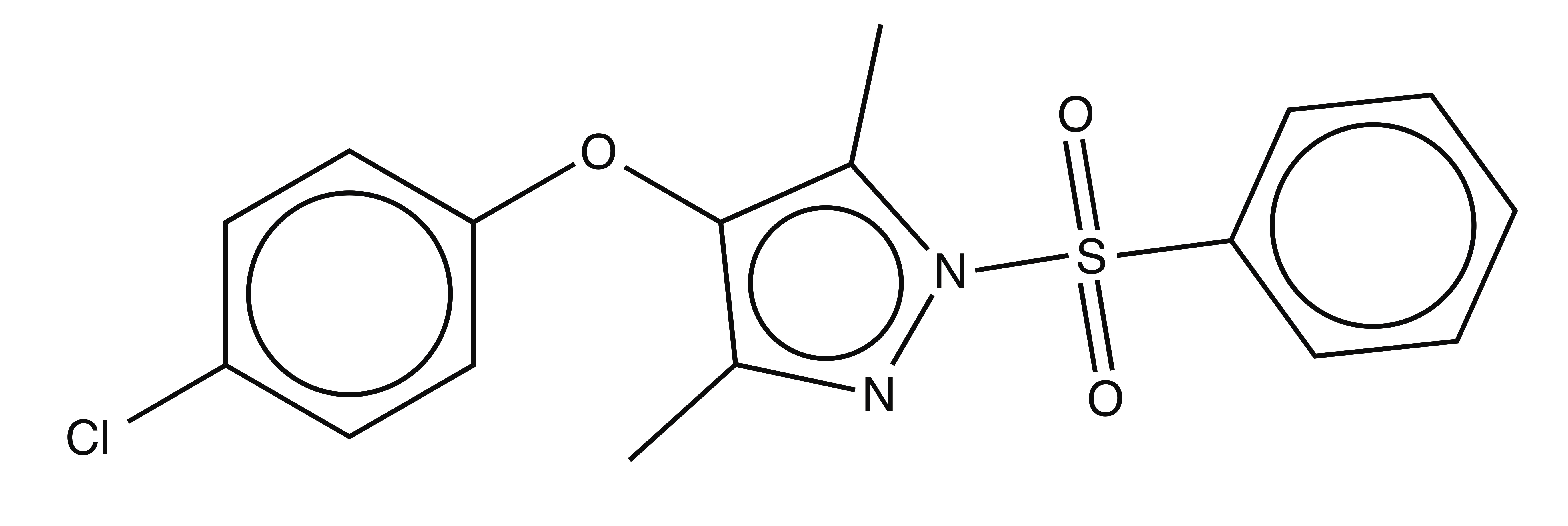 | N/A | N/A |
| D18 CID 2747279 | 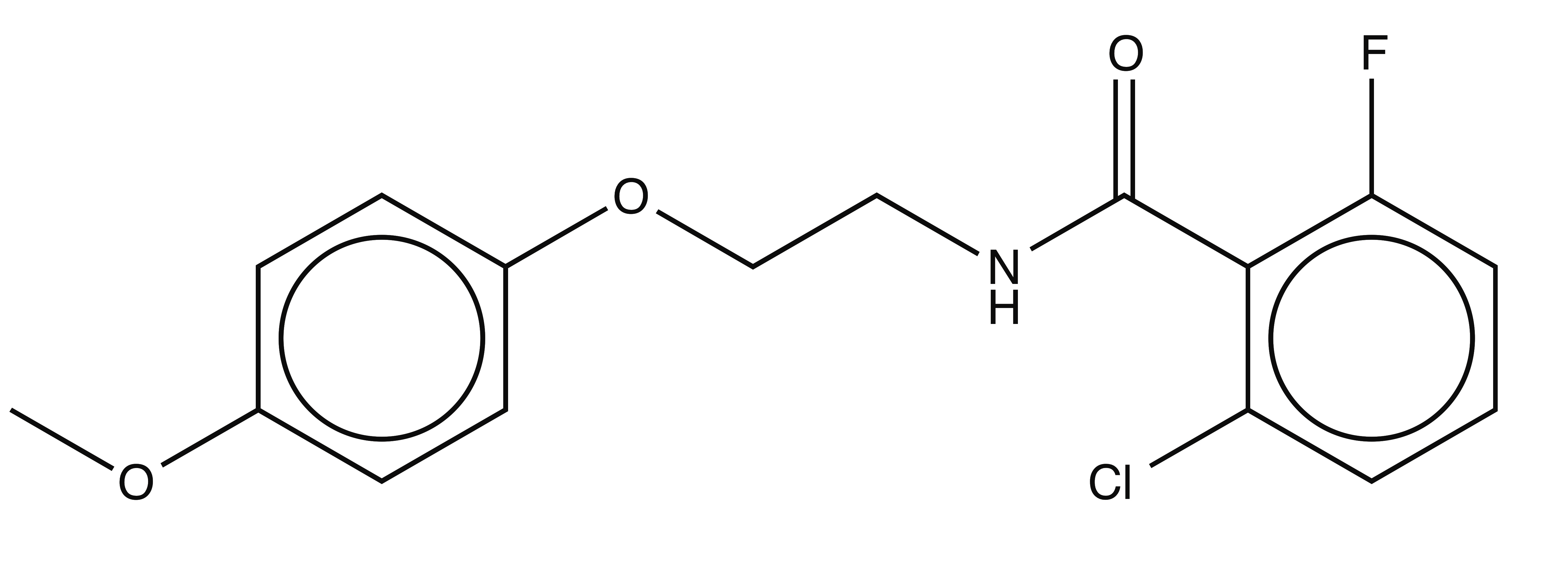 | Cluster 10 | 0.62  WACT11 |
| D19 CID 2747322 | 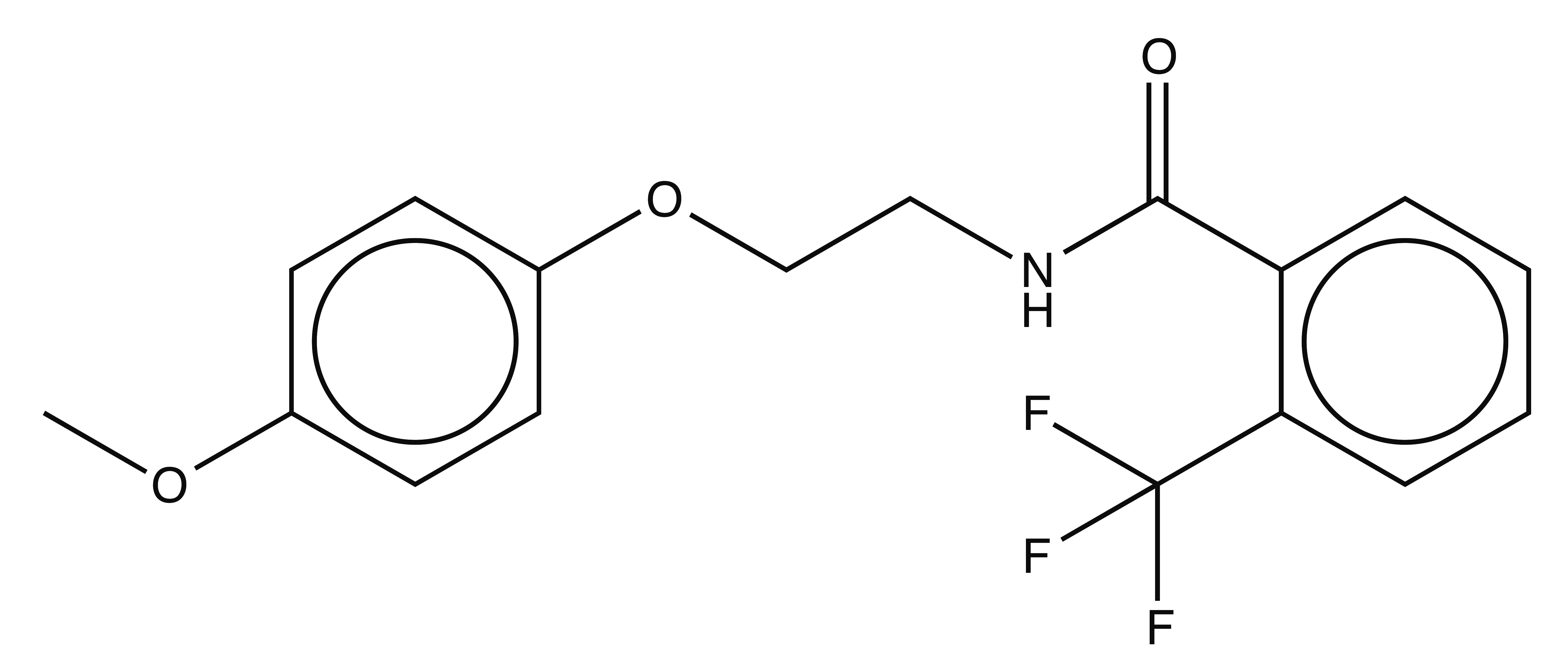 | Cluster 10 | 0.67  WACT11 |

**Supplemental Table 3.** Nematode hit compounds overlap with Burns study.
