# Supplementary material for: Using C. elegans Forward and Reverse Genetics to Identify New Compounds with Anthelmintic Activity
Source: PLoS Negl Trop Dis. 2016 Oct 18;10(10):e0005058. doi: 10.1371/journal.pntd.0005058 (PMC5068747; doi:10.1371/journal.pntd.0005058)
Supplement: S1 Software — (ZIP) [file pntd.0005058.s008.zip › ScannerProjectUserDocument.doc]

**Scanner Project User Document**

**Written by Mike Simpson**

**July 2, 2014**

**INDEX**

Installation	2

ScannerProject.jar	2

ScannerProject.ini	2

ScannerTemplates Folder	2

Setup and Initialization	3

Initialization File	3

Excel Reports	3

Configuring Experiment	4

Experiment Type	4

Single Scan	4

Multiple Scans	4

Well Size	4

Selecting Images	5

Single Scan	5

Multiple Scans	5

Aligning Images	6

Viewing Results	7

# Installation

The installation of the scanner project only requires that the ScannerProject.jar, ScannerProject.ini, and ScannerTemplates folder are all stored in the same directory.

## ScannerProject.jar

This file is the executable used to run the project.

## ScannerProject.ini

All of the configuration settings are stored in this file.

## ScannerTemplates Folder

This folder contains the excel files used as templates for reporting.

Setup and Initialization

## Initialization File

Any text editor can be used to view the file. Settings such as the score cut-off for red/yellow/green as well as the default well sizes and well positions for each plate size can be found in this file. There is a description above each setting to describe their meaning.

## Excel Reports

There is an individual file for each plate size/experiment type combination. The first tab of each excel file is where the raw data is stored from each scan, and should not be altered. Each of the other tabs can contain any representation of the data that excel provides. New tabs can be created as well. When the image processing is completed in the application, a copy of the appropriate excel file is made with the raw data inserted. You will then be prompted to save the new copy, with all of the template file information pre-loaded.

# Configuring Experiment

Select the experiment type and well size desired, and then click *Next*.

## Experiment Type

### Single Scan

A *single scan* experiment mean that only a single pair of scans will be processed. This scan should be used to determine relative degree of movement in each well.

### Multiple Scans

*Multiple scans* should be selected when there are several pairs of scans that have been taken on a single plate. For example: a lifespan assay that had been scanned every hour. These scans will be used to show how the movement has changed from scan to scan.

## Well Size

The well size is the standard number of wells per plate.

Selecting Images

This interface is used to select the images that are to be processed. The number of images you need to select depends on the experiment type that was selected on the previous screen. Click *Search* to find the scanned images. You can click *Previous* to go back, or hit *Next* once the images have been selected.

## Single Scan

If *Single Scan* was selected on the previous screen, then the two paired images must be selected.

## Multiple Scans

If Multiple Scans was selected on the previous screen, then each of the paired images must be selected. They also must be selected in the order they are to be processed.

Aligning Images

This interface is used to identify to the ScannerProject the location of the wells. The four corners of the plate are represented in this image. Each of the image transformations is done with respect to the *Top Left Well*, so that well should be aligned first before rotating or changing the row and column size. You can shift the image, change the row and column size, as well as rotate the image in order to align the wells. Each of the image transformations is done with respect to the *Top Left Well*, so that well should be aligned first before rotating or changing the row and column size. The resulting changes are recorded in the bottom right of the interface, and those number can be used to enter into the initiation file to be the new default if the same image transformations are always needed.

If more than a single pair of images has been selected, hitting the *Previous* and *Next* buttons will cycle through them. If the plate has not been moved during each of the scans, then it will not be necessary to individually align them. Select *Set for all alignments* and then click the *Next* button in order to set each of the other plates to those same settings.

Viewing Results

The results for each of the paired images are viewed individually. The *Previous* and *Next* buttons can be used to cycle through the results of each scan. The individual wells can be clicked to bring up the *Swap Images* interface in order to actually view the scanned image of the wells. The number in each well represents the number of changed pixels detected. The higher the number of changed pixels, the more movement occurred within the well.

# Swap Images

In order to verify the results of the image processing, each well can be clicked individually to compare the wells between the first, second, and difference image. The first and second images are each of the paired images in the scan. The difference image consists of each of the changed pixels that were detected.

|  |  |  |
| --- | --- | --- |
